# Experimental Genome-Wide Determination of RNA Polyadenylation in *Chlamydomonas reinhardtii*

**DOI:** 10.1371/journal.pone.0146107

**Published:** 2016-01-05

**Authors:** Stephen A. Bell, Chi Shen, Alishea Brown, Arthur G. Hunt

**Affiliations:** 1 Department of Plant and Soil Sciences, University of Kentucky, Lexington, Kentucky, United States of America; 2 Division of Computer Science, Kentucky State University, Frankfort, Kentucky, United States of America; Donald Danforth Plant Science Center, UNITED STATES

## Abstract

The polyadenylation of RNA is a near-universal feature of RNA metabolism in eukaryotes. This process has been studied in the model alga *Chlamydomonas reinhardtii* using low-throughput (gene-by-gene) and high-throughput (transcriptome sequencing) approaches that recovered poly(A)-containing sequence tags which revealed interesting features of this critical process in Chlamydomonas. In this study, RNA polyadenylation has been studied using the so-called Poly(A) Tag Sequencing (PAT-Seq) approach. Specifically, PAT-Seq was used to study poly(A) site choice in cultures grown in four different media types—Tris-Phosphate (TP), Tris-Phosphate-Acetate (TAP), High-Salt (HS), and High-Salt-Acetate (HAS). The results indicate that: 1. As reported before, the motif UGUAA is the primary, and perhaps sole, *cis*-element that guides mRNA polyadenylation in the nucleus; 2. The scope of alternative polyadenylation events with the potential to change the coding sequences of mRNAs is limited; 3. Changes in poly(A) site choice in cultures grown in the different media types are very few in number and do not affect protein-coding potential; 4. Organellar polyadenylation is considerable and affects primarily ribosomal RNAs in the chloroplast and mitochondria; and 5. Organellar RNA polyadenylation is a dynamic process that is affected by the different media types used for cell growth.

## Introduction

RNA processing in eukaryotic organisms is an intricate process comprised of multiple events that all appear to take place simultaneously before an mRNA is exported to the cytoplasm where translation to protein takes place [1–3]. Primarily, the major events that comprise post-transcriptional RNA processing include 5’-end methylguanosine capping, intron/exon splicing, and 3’-end polyadenylation. Processing at the 3’-end of pre-mRNA transcripts has been well documented in animals, plants, and fungi where many similarities have been identified in addition to key differences amongst these kingdoms [4, 5]. While this process has often been dubbed ubiquitous and even overlooked in terms of importance, demonstrations of its links to critically important cellular fates continue to emerge. For example, alternative polyadenylation has been linked to oncogene activation in human cancer cells as well as flowering time in plants [6, 7].

Despite the great progress that has been made in understanding polyadenylation in plants, animals, and fungi, considerably less is known about the specifics of this process in algae. Historically, the green alga *Chlamydomonas reinhardtii* has attracted the attention of countless scientists and has contributed significantly to the fields of photosynthesis and cell motility [8, 9]. While Chlamydomonas still holds an important place in these areas, it has gained momentum as a host for biotechnological applications over the past 25 years; these include using Chlamydomonas as a platform for the renewable production of hydrogen gas as well as therapeutic proteins and high-value small molecules (reviewed in [10–12]).

Despite this interest, however, and the inherent benefits of Chlamydomonas (generally recognized as safe, fast growing, photosynthetic, sequenced genome), bioengineering of this organism has only been successful in a context dependent fashion. Whereas many successful accounts have been made for the expression of transgenes from the chloroplast genome [13, 14], particular difficulties have been faced during efforts to bioengineer the metabolism of Chlamydomonas *via* nuclear transgenes although progress has been made to better understand the limitations [15, 16]. To date, the best example of expressing catalytically active, non-selectable enzyme from the nuclear genome was done by Rasala et al. where the ble coding sequence, 2A peptide bond skipping sequence, and coding sequence for a xylanase were combined as a single fusion construct [17]. In the presence of zeocin, Chlamydomonas was forced to express both the ble resistance enzyme (to survive) and as a by-product, the xylanase which was catalytically active. Whether or not this approach will result in significant manipulations of metabolism in Chlamydomonas has yet to be demonstrated to the best of our knowledge.

Interestingly, problems expressing nuclear transgenes also existed during early efforts to engineer higher plants [18] and were ultimately overcome by researchers who demonstrated the importance of having the correct *cis*-elements in the 3’-end of gene constructs [19–22]. These *cis*-elements, important for the proper polyadenylation of resulting mRNA species, were ultimately responsible for inefficient transgene expression in higher plants. Given this and the observations that have been reported for Chlamydomonas (as well as firsthand attempts), we surmised one possibility for the lack of success in bioengineering metabolism directed by nuclear transgenes, could be the result of poorly understood gene processing events. Hence, we set out to analyze global polyadenylation practices by Chlamydomonas as a function of culturing conditions that have commonly been used by researchers attempting to engineer and grow this alga under laboratory conditions.

## Materials and Methods

### Media

The media recipes used for all experimental work are as follows and can be found on the Chlamy Center website (http://www.chlamy.org/media.html). Tris-Phosphate (TP) media (originally described by Gorman and Levine [23]) was prepared by mixing 2.5 g Tris, 25 mL Solution 1 (per liter: 15 g NH_4_Cl, 4 g MgSO_4_ • 7H_2_O, 2 g CaCl_2_ • 2H_2_O), 0.375 mL Solution 2 (per 100 mL: 28.8 g K_2_HPO_4_, 14.4 g KH_2_PO_4_) and 1 mL of Hutner’s trace elements (see http://www.chlamy.org/trace.html for recipe), adjusting the pH to 7 then the final volume to 1 L with Milli-Q H_2_O. To prepare Tris-Phosphate-Acetate (TAP) media, the same recipe for TP was followed except 2 g of sodium acetate trihydrate was added. High-Salt (HS) media (originally described by Sueoka [24]) was prepared by mixing 5 mL of Solution 1 (per liter: 100 g NH_4_Cl, 4 g MgSO_4_ • 7H_2_O, 2 g CaCl_2_ • 2H_2_O), 5 mL of Solution 2 (per 100 mL: 28.8 g K_2_HPO_4_, 14.4 g KH_2_PO_4_), and 1 mL of Hutner’s trace elements, adjusting the pH to 7 then the final volume to 1 L with Milli-Q H_2_O. To prepare High-Salt-Acetate (HAS) media, the same recipe for HS was followed except 2 g of sodium acetate trihydrate was added. All media types used were sterilized by autoclave immediately after preparation and stored at room temperature until needed.

### Strains and culture conditions

The wild type *Chlamydomonas reinhardtii* strain CC-1690 (obtained from Chlamy Center) was used for all experiments described in this work. Generally, this strain was maintained on TAP agar slants at room temperature, which were restreaked at approximately one-month intervals. Liquid starter cultures were generated as follows: CC-1690 cells from a fully grown slant culture (2–4 weeks old) were transferred to 10 mL of TP or HS media in a 50 mL flask using a 1 mL serological pipet. These cultures were then grown for approximately 2 weeks in an orbital shaker at 25°C with 150 rpm shaking under continuous lighting (fluorescent, ~325 lux). From these cultures, 5 mL was used to inoculate 50 mL of TP or HS media in a 250 mL flask (foam stoppers were used instead of aluminum foil to minimize blockage of light) which was grown for 5 days at 25°C with 150 rpm shaking under continuous lighting. 2 mL of this 5-day-old 50 mL TP starter culture was used to inoculate 100 mL of TP or TAP in a 500 mL flask with a foam stopper; triplicate cultures for each media type were started at the same time. The same was done for 100 mL triplicate cultures in HS and HAS media, but using the 50 mL HS starter culture instead. After five days of growth, cells from each culture were collected by centrifugation, the media was removed, and the cell pellets were stored at -80°C until RNA was extracted.

### Chlamydomonas RNA Isolation

All RNA was isolated using TRI REAGENT^®^ from Molecular Research Center, Inc. according to their protocol. Typically, RNA for a given sample was isolated as follows. 50–100 mg of frozen cells were weighed and resuspended in 1 mL of TRI REAGENT^®^. A homogeneous mixture was obtained by vigorously vortexing, which was then incubated at room temperature for 5 minutes. Next, 200 μL of chloroform was added, vortexed for 15 seconds, then incubated at room temperature for 15 minutes. Phase separation was accomplished by centrifuging at 12,000 x g for 15 minutes at 4°C. The aqueous (upper) phase was transferred to a new tube being very careful not to touch or remove any of the interphase layer. 250 μL of isopropanol was added to this along with 250 μL of salt solution (0.8 M sodium citrate, 1.2 M NaCl), mixed well, and incubated at room temperature for 10 minutes. The RNA was pelleted by centrifuging at 12,000 x g for 8 minutes at room temperature, the supernatant was removed, and 1 mL of cold 75% ethanol was added, then vortexed. Again, the RNA was pelleted by centrifuging at 12,000 x g for 5 minutes at room temperature and the ethanol was removed. The pellet was air dried for 5 minutes, dissolved in 30 μL of nuclease free water, and heated to 65°C for 5 minutes. Quantity and quality measurements were taken using standard spectrometric techniques in addition to visualization on a 0.8% agarose gel to assess intactness.

### Poly(A) tag library preparation and sequencing

So-called poly(A) tags, short cDNAs that query the mRNA-poly(A) junction, were prepared following Method B1 as described in Ma et al. [25], using between 1 and 5 μg of total RNA per library. These libraries were sequenced on a MiSeq instrument and the sequencing data processed using the pipeline detailed in S1 File.

## Results

### Preparation and sequencing of poly(A) tag libraries prepared from Chlamydomonas

To study poly(A) site choice in Chlamydomonas, the wild type strain CC-1690 was grown in four different media types: Tris-Phosphate (TP), Tris-Phosphate-Acetate (TAP), High-Salt (HS), and High-Salt-Acetate (HAS). Triplicate cultures for each media type were grown under constant light at 25°C with 150 rpm orbital shaking for five days until cells were collected and RNA was isolated. Under these conditions and at the time of harvest, the cells were early in an active growth phase (S1 Fig); the TAP culture was at a decidedly higher density than the other three, but at a stage in the growth process reflective of rapid growth (as opposed to stationary phase). These parameters were chosen because they have often been used as the initial laboratory conditions by researchers attempting to bioengineer Chlamydomonas. Hence, we envisioned that this subset of culturing conditions would provide a rich, diverse, but still relevant data set regarding important effects on polyadenylation and gene expression in this alga under typical laboratory conditions.

So-called poly(A) tags (PATs), short cDNAs that contain the mRNA-poly(A) junction, were prepared from the isolated RNA following the protocol described in Ma et al. and Pati et al. [25, 26]. Following PAT sequencing, the data was processed as described in S1 File. Briefly, the raw sequences were demultiplexed using the bar codes built into the reverse transcription primers and the remaining tracts of oligo-dT as well as segments of the sequencing adapters were removed (present in cases where short inserts were sequenced). The sequences were then mapped to the Chlamydomonas genome (Creinhardtii_281_v5.5); the results of this exercise are summarized in S2 File. Mapped tags and the corresponding genome coordinates were recovered followed by “reduction” of the tags to one-base coordinates that corresponded to the 5’-end of the tag (or 3’-end of the corresponding RNA). This collection of sequences and coordinates were then used for the analyses described in the following sections.

To evaluate the reproducibility of the library preparations and the representation of the PATs with respect to genes that should be expressed in cells grown under the four conditions, the trimmed PATs were used to estimate relative gene expression levels in the four growth conditions. These results are compiled in S3 File. The library-by-library comparison for gene expression values indicates a high degree of correlation between libraries, with only one library being somewhat different from the others (S2 Fig). 2,551 genes (of a total of 17,721 annotated genes; 14.39%) showed differential expression in at least one of the four growth regimens (S3 File) suggesting that these commonly employed media types can have important effects on gene expression in Chlamydomonas.

**Fig 1:**
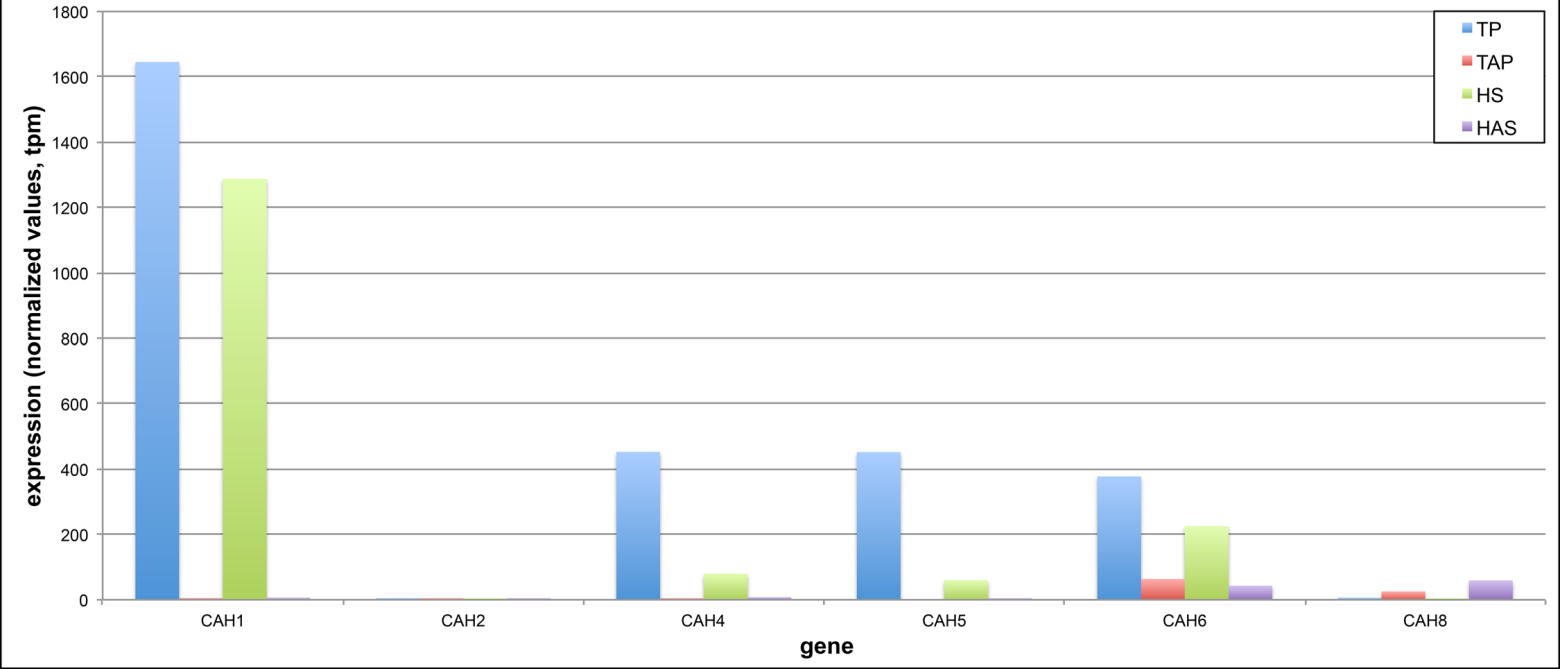
Comparative expression analysis of Chlamydomonas carbonic anhydrase (CAH) genes. Cells grown in two different media types with and without acetate (TP–Tris-Phosphate, TAP–Tris-Phosphate-Acetate, HS–High-Salt, HAS–High-Salt-Acetate) were profiled for poly(A) site choice *via* construction of PATs followed by mapping of the tags to the annotated Chlamydomonas genome. Total PAT counts for each gene were normalized using the values from triplicate samples grown in the four different media types and presented as tags per million (tpm) for each of the genes shown. Phytozome 10.3 gene IDs for the CAHs used are as follows: CAH1 –Cre04.g223100, CAH2 –Cre04.g223050, CAH4 –Cre05.g248400, CAH5 –Cre05.g248450, CAH6 –Cre12.g485050, CAH8 –Cre09.g405750.

Of particular interest among the annotated, differentially expressed genes were those involved in the carbon concentrating mechanism (CCM) for Chlamydomonas. This process has not only been well studied and therefore had many of the key players characterized (reviewed in [27]), but it also presented a good opportunity to examine differential gene expression as a function of media type, primarily in the plus/minus acetate media. Chiefly, the genes involved include a series of carbonic anhydrases (CAH) that localize to specific cellular compartments, several (proposed) transporters and channels thought to be responsible for translocation of the carbon forms involved (CO_2_ and HCO_3_^-^), and a few regulators of the pathway (reviewed in [27]). Given the media types used, one might expect that CCM-related genes would be expressed at higher levels for algae grown in media that did not contain acetate. These algae would be solely reliant upon CO_2_/HCO_3_^-^ dissolved in the media as opposed to the algae grown in the presence of acetate, which could readily be taken up and utilized as the main carbon source. As expected, the majority of carbonic anhydrase genes known to be associated with the CCM in Chlamydomonas were expressed at higher levels for algae that were grown in the absence of acetate (Fig 1).

### Genome-wide distribution of poly(A) sites in Chlamydomonas

The collection of PATs from the twelve libraries define numerous possible poly(A) sites although many of these could be the result of inadvertent and rare priming events, as opposed to authentic priming at the poly(A)–mRNA junction. To focus on high-confidence sites, only sites that were represented by 10 or more individual tags in the collection of libraries were retained for subsequent analyses. This process yielded 46,308 individual poly(A) sites (or PAS; these are listed in S4 File); these sites were defined by 94.6% of all of the poly(A) tags and thus represent the overwhelming majority of gene expression in the study. The vast majority of these sites (40,996, or >88%) were of the same orientation as their associated annotated genes. Of these, more than 96% mapped to annotated 3’-UTRs, or within 25 nts of the 3’-ends of annotated 3’-UTRs (S4 File). Approximately 2% of sites mapped to within protein-coding regions, while 0.84% and 0.76%, respectively, mapped to 5’-UTRs or introns. 0.2% of PATs mapped to genomic regions whose annotations are ambiguous (usually due to the overlap of features of alternative transcripts). Of the sense-oriented PATs that mapped to annotated regions of the genome, more than 97% mapped to annotated 3’-UTRs, or within 25 nts of these regions (S4 File).

**Fig 2:**
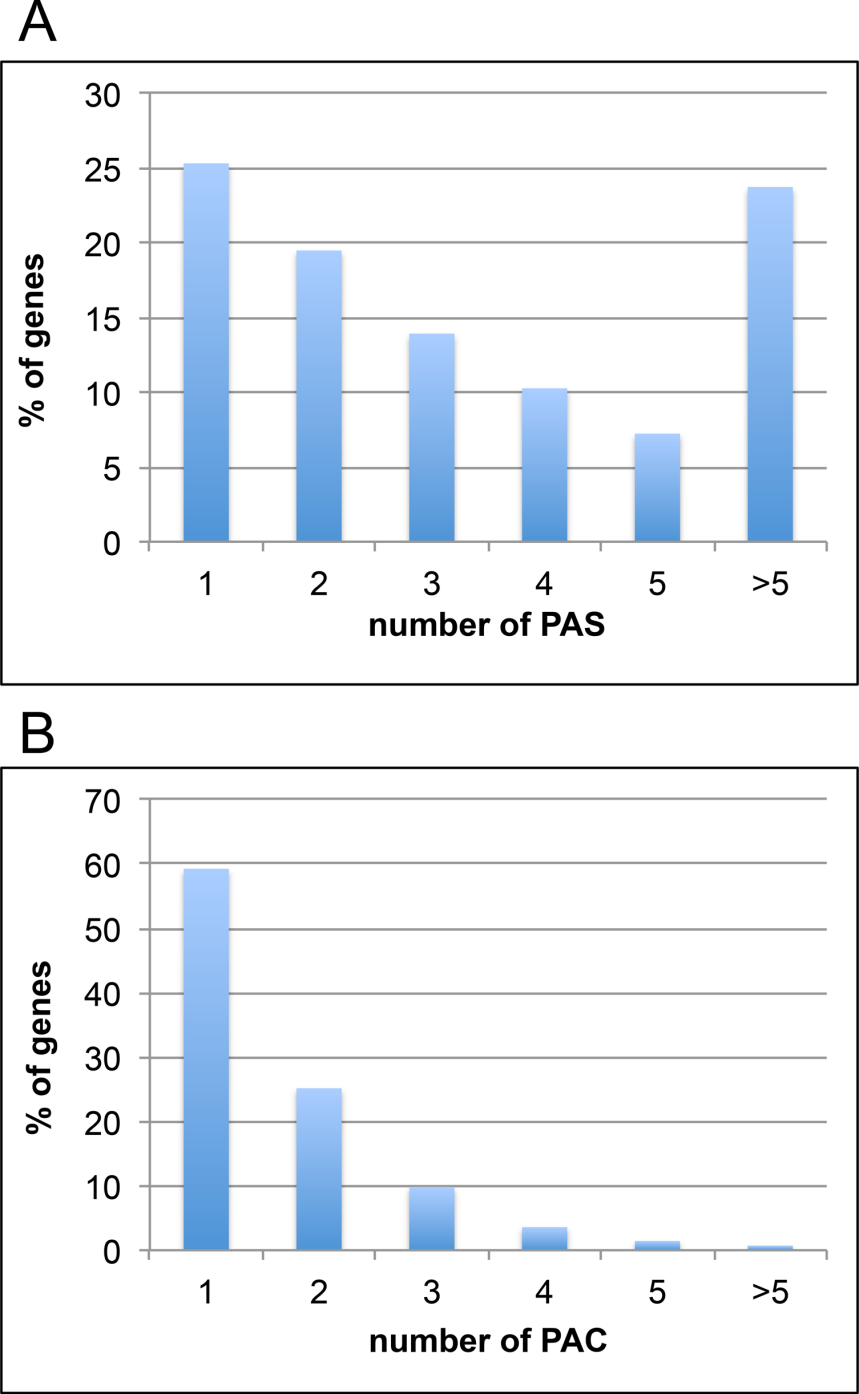
Chlamydomonas genes with multiple poly(A) sites. (A) Fraction of all genes with 1, 2, 3, 4, or >5 individual poly(A) sites (PAS). Total number of PAS = 41,048. (B) Fraction of all genes with 1, 2, 3, 4, 5, or >5 poly(A) site clusters (PACs). Total number of PACs = 19,674.

The 40,996 sense-oriented poly(A) sites mapped to 9,232 Chlamydomonas genes. 75% of these genes had at least two poly(A) sites, and almost 25% had more than five (Fig 2A). However, visual inspection suggested that many of these poly(A) sites occurred as clusters, similar to what has been observed in higher plants [28, 29]. Accordingly, the individual poly(A) sites were clustered together (before filtering for numbers of PATs) such that sites within 24 nts of each other were grouped together into single poly(A) clusters (PACs). PACs that were defined by fewer than ten PATs were removed followed by identification of the sense-oriented PACs. This exercise resulted in a collection of 22,410 PACs (S5 File) defined by 98.4% of all mapped poly(A) tags. 19,574 of these PACs mapped to 11,887 annotated genes and had the same orientation. As was the case for individual PAS (S4 File), almost all (92%) of the sense-oriented PACs mapped to 3’-UTRs or the adjacent 25 nts (S5 File). Only 8% of PACs mapped to genomic regions (introns, 5’-UTRs, protein-coding regions) that, if chosen for polyadenylation, could affect mRNA functionality. Of the 11,887 genes with PACs, almost 60% had a single PAC, and almost 95% had three or fewer PACs (Fig 2B). These results indicate that individual poly(A) sites tend to occur in clusters, and that most Chlamydomonas genes have but a single PAC that is situated within annotated 3’-UTRs.

### Sequence elements associated with different classes of poly(A) sites

**Fig 3:**
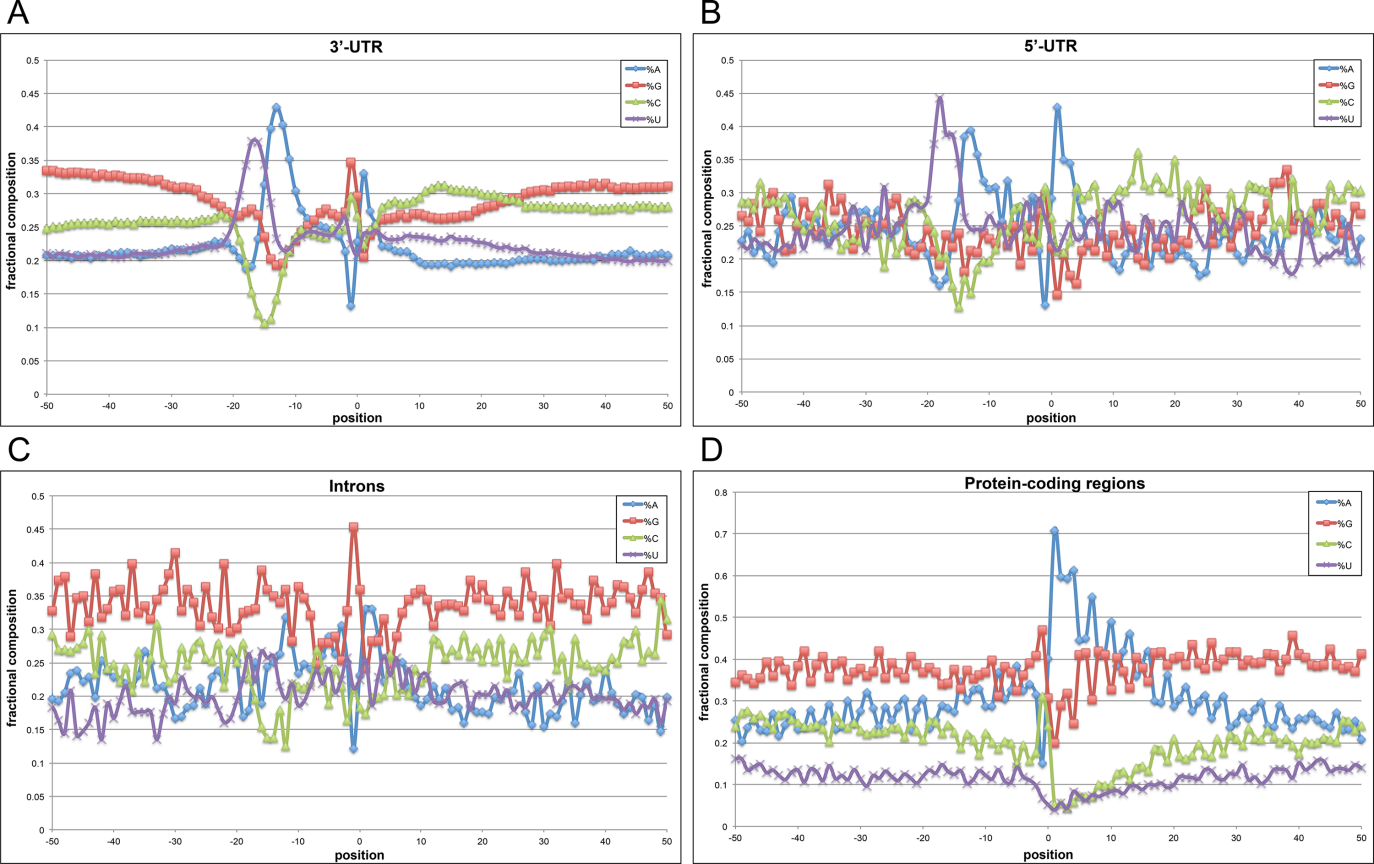
Position-by-position base composition of poly(A) sites for different genic regions in Chlamydomonas genes. The panels shown depict the following genic regions: (A) 3’-UTRs, (B) 5’-UTRs, (C) introns, and (D) protein-coding regions. Y-axis values are the fractional nucleotide content at each position (plotted along the x-axis) with individual traces color-coded as indicated in the legend. On the x-axis, “0” denotes the actual cleavage/polyadenylation site–negative values represent positions that are 5’ (upstream) of the poly(A) site and positive values are positions 3’ (downstream) of the poly(A) site. Number of poly(A) sites used for each analysis are as follows: A = 39,412, B = 343, C = 311, and D = 840.

**Fig 4:**
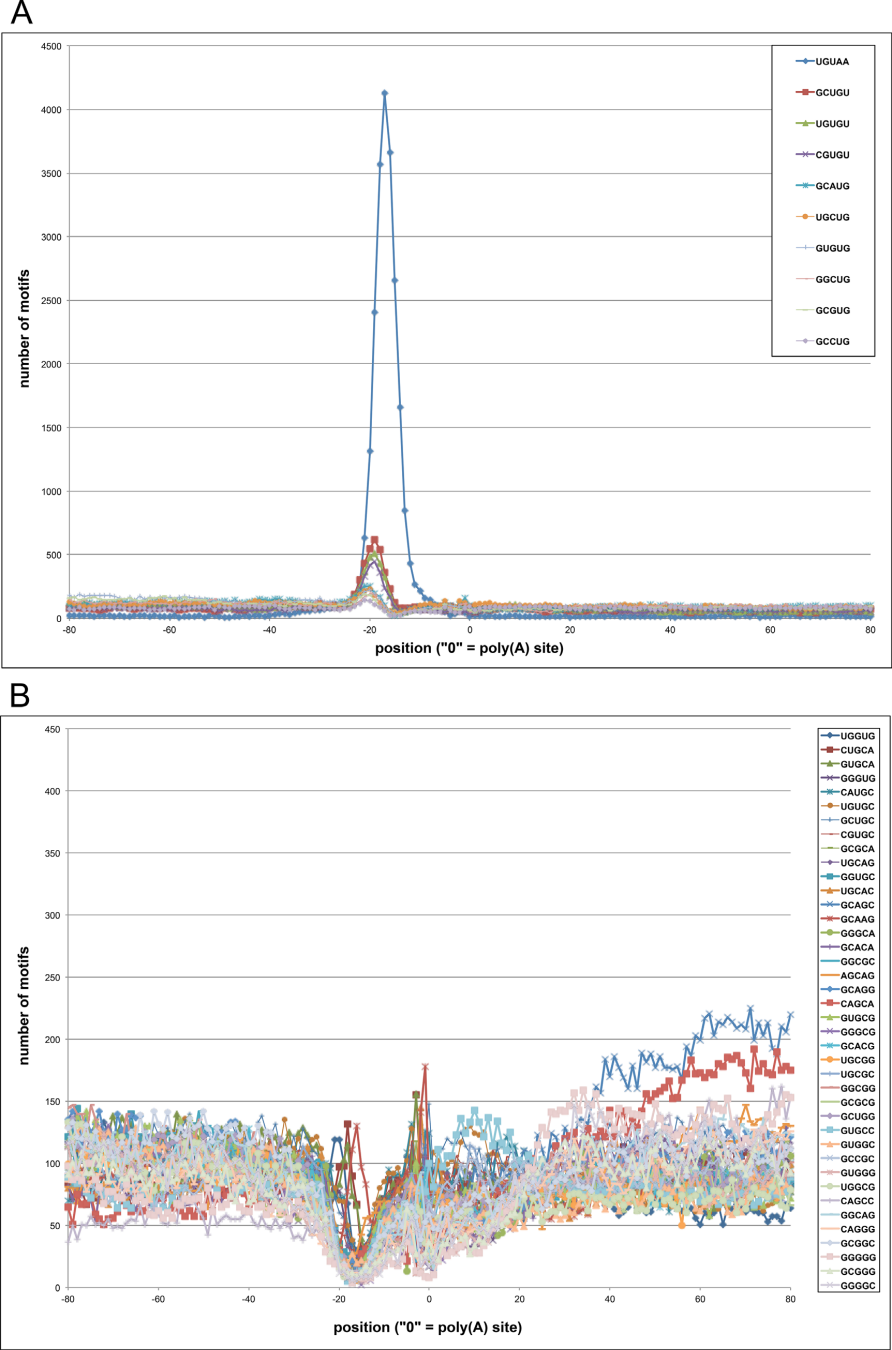
Motif analysis of regions surrounding 3’-UTR-situated poly(A) sites in Chlamydomonas. The occurrences of 5 nt motifs was determined using SignalSleuth2 [51]. The relative position of the motif is given on the x-axis, with the poly(A) site being set as “0”; in these plots, regions extending from 80 nts 5’ (upstream) to 80 nts 3’ (downstream” from the poly(A) sites are shown. The numerical count of each motif is given on the y-axis. The plot shows the distributions of the 50 most-abundant motifs, which are listed in the legend embedded on the right. The number of sites used for this analysis was 39,415. (A) Profiles of the 10 most abundant motifs. The individual motifs are noted in the caption in the upper right corner. (B) Profiles of the next 40 most abundant motifs. The individual motifs are noted in the caption on the right. Note that the y-axis scale for panel B is 10% of that for panel A.

Previous reports have suggested that Chlamydomonas has a distinctive polyadenylation signal (UGUAA) [30–32], which is quite different from the canonical motifs associated with polyadenylation in mammals, yeast, and plants [4, 5]. To explore this issue further, sequences associated with several different classes of poly(A) sites as defined by the poly(A) tags generated in this study were characterized. Two analyses were performed. In one, the general nucleotide composition surrounding each class of site was determined (Fig 3). In the other, specific sequence motifs associated with distinctive positions with respect to each class of site were identified and displayed (Fig 4).

Shown in Fig 3A, the general nucleotide composition surrounding poly(A) sites situated in annotated 3’-UTRs had a distinctive profile, with two features. The first feature was a distinctive peak of U- and A- richness between 10 and 20 nts 5’ (or upstream) of the poly(A) site. This coincides with the highly conserved UGUAA signal that has been noted by others and was also apparent in the motif analysis (Fig 4). The second feature was a trend towards G+C at the poly(A) site itself, flanked by a peak of A. Interestingly, while the latter trend was unmistakable, it did not reflect the presence of a conserved motif at the poly(A) site (Fig 4). Similar trends, especially in the -10 to -20 region, were seen with sites situated in 5’-UTRs (Fig 3B). The result obtained from an analysis of intron-situated sites did not display the UGUAA motif in the -10 to -20 region (Fig 3C), suggesting that these sites may be different in nature from those found in the UTRs. Important to note here, however, is that this assignment is more tentative than others due to the low prevalence of such sites. Poly(A) sites situated within protein-coding regions displayed a distinctly different pattern and were typically located within A+G-rich regions (Fig 3D). This trend is very similar to what has been reported for coding region-situated poly(A) sites in higher plants [29, 33].

The results of searches for over-represented motifs in the vicinities of 3’-UTR-situated poly(A) sites are shown in Fig 4. This analysis demonstrated a clear enrichment for the UGUAA motif between 10 and 20 nts upstream of the poly(A) site. This motif was found in this location in 58% of all the sites situated in 3’-UTRs, and related motifs (with four of the five positions matching the UGUAA consensus) are found in 95% of all such sites. The remaining sites almost always were variants of this motif that differed in only two positions, or were located outside of the -10 to -20 window. This was reflected in the decided nucleotide composition of these sites (S3 Fig).

Poly(A) sites situated within 5’-UTRs possessed the same UGUAA motif that was observed in sites located in 3’-UTRs (S6 File). No such association was seen for poly(A) sites situated in introns (S6 File). These sites did possess two possible motifs (GGGGG and UUUUU) but the small sample number makes a firm assessment tenuous. CDS-situated sites also lacked the UGUAA motif (S6 File) and instead, were enriched in motifs that reflect the high A+G content of these sites (Fig 3D).

Some 5,300 poly(A) sites, defined by 3 million PATs, map to as-yet unannotated regions of the Chlamydomonas genome, or to regions annotated as other than protein-coding (S4 File). These sites have the same general base composition (S4A Fig) and motif bias (S4B Fig) as do sense-oriented sites that map to 3’-UTRs. This suggests that these are authentic sites and not the products of inadvertent priming by reverse transcriptase or artifactual over-amplification during the PCR steps in library preparation. These sites, however, were much more disposed to possess an additional motif (UUUUU) near or at the cleavage/polyadenylation site. Of these sites, 835 (16%) fell within 100 bp of annotated 3’-UTRs that had been extended by 25 nts and were in the same orientation as the PAS (S7 File); these probably represent 3’-UTR extensions for the respective genes. The remaining 84% likely define as-yet unidentified genes.

### Alternative poly(A) site usage in Chlamydomonas

As indicated in Fig 2, about 40% of Chlamydomonas genes possessed two or more PACs. While somewhat lower than what has been observed in other eukaryotes, this number still allows for a considerable potential for alternative poly(A) site choice. To study this, poly(A) site usage in cells grown in the four different media types was compared and individual PACs whose usage varied in statistically-significant ways were identified. The DEXSeq package, developed to study alternative splicing [34], was used for this analysis. 273 individual PACS (of more than 19,500) were found to exhibit significantly different usage under one or more of the four conditions studied (S8 File). These differential site choices impacted 172 genes, most (76%) of which were present within 3’-UTRs (S8 File). 5% of the differentially utilized sites mapped to 5’-UTRs that were within 250 bp of a nearby, upstream gene with the same orientation (S8 File); these sites are probably flagged as alternatively processed because of altered expression of the upstream gene.

For the most part, sites located outside of 3’-UTRs represented low-abundance transcript isoforms, and the changes in usage did not have a significant effect on the abundance of the major mRNA isoform associated with the affected gene (this may be gleaned from inspection of S8 File). In some cases, though, the major isoforms differed in different conditions (examples are shown in S5 Fig).

**Fig 5:**
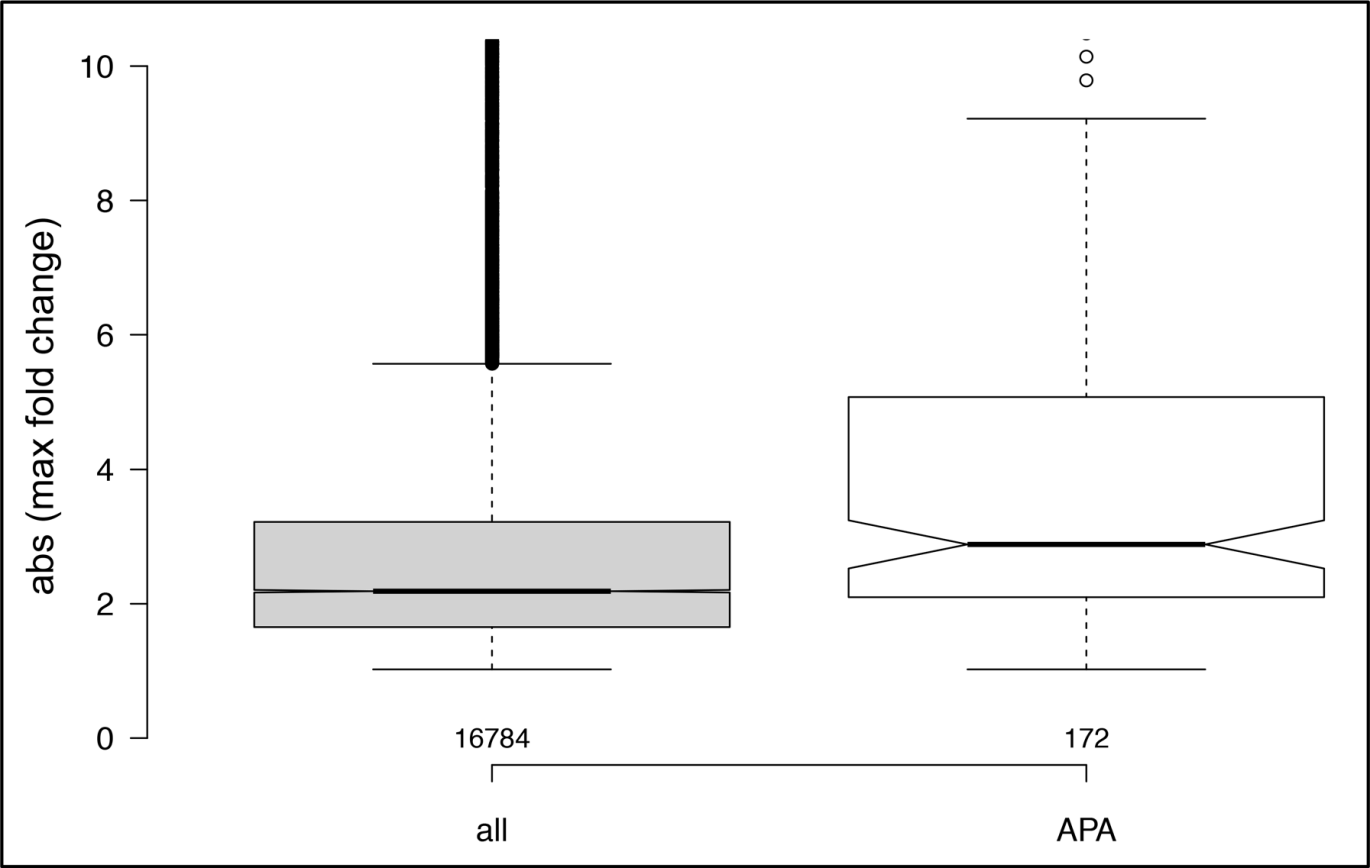
Box plot comparing the changes in expression of genes affected by alternative polyadenylation (“APA”) with the changes of all genes expressed in at least one of the four treatments (“all”). Numbers of genes: for “all” = 16,784, for “APA” = 172. Values on the y-axis denote the absolute value for the maximum fold-change for a given gene in the pairwise comparisons that were performed.

The four growth regimens studied here incite significant changes in the expression of numerous genes (S3 File). To study possible associations between altered poly(A) site choice and differential gene expression, the changes in expression in the set of 172 genes affected by alternative poly(A) site choice were compared with the range of such changes of all genes. The results (Fig 5) show at best modest association of altered gene expression with alternative poly(A) site choice.

### Polyadenylation of organellar RNAs

**Fig 6:**
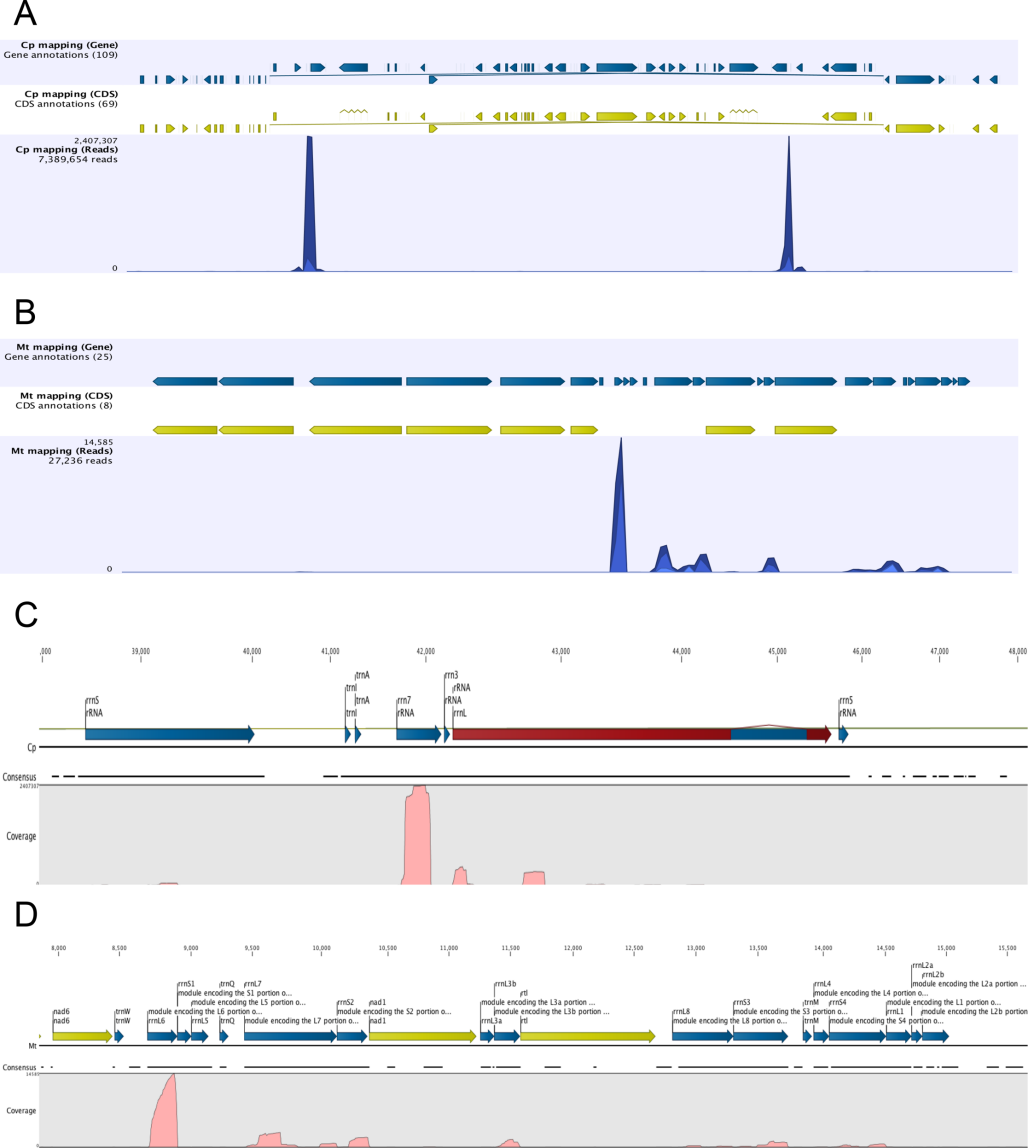
Genome browser views of PAT mappings to the Chlamydomonas chloroplast and mitochondrial genomes. Panels A and B depict the results of mappings to the complete chloroplast and mitochondrial genomes, respectively, while C and D show mappings to the rRNA-encoding regions in the chloroplast and mitochondria, respectively (in the case of panel C, only one of the two chloroplast rRNA-encoding regions is shown). In panels A and B, the uppermost plots denote the locations of annotated genes, the middle plots show the locations of annotated protein-coding regions, and the lower plots are bar graphs representing the tag abundance across the genome. In panels C and D, the uppermost plots depict details of the gene annotations, the middle line (“consensus”) shows the overall extent of contiguous tag mappings, and the lower plots shows the relative tag abundances across the depicted genomic regions.

The process of RNA polyadenylation in eukaryotes is not limited to the nucleus in eukaryotes, but also affects organellar RNAs [35]. Since the RNA samples used for library preparation were derived from whole cells, and not fractionated extracts, the representation of organellar RNAs in the PAT libraries was examined (S2 File). A relatively large number of PATs were found to map to the chloroplast genome, and a much smaller but nonetheless significant number to the mitochondrial genome. Interestingly, in both compartments, the overwhelming majority of PATs mapped to ribosomal RNAs (Fig 6A and 6B). In the mitochondria, almost all PATs mapped to various rRNA-encoding regions of the genome (Fig 6B). The distribution of PATs along the rRNA transcription units in the two organelles was not random, but localized to a few regions. Thus, in the chloroplast, most of the PATs mapped to the rrn7 locus, and seemed to define polyadenylation events occurring within the corresponding RNA (Fig 6C). Other events could be inferred near the 5’-end of the 23S RNA and the middle of the 16S RNA (Fig 6C). In mitochondria, most of the rRNA-associated PATs mapped to the 3’-end of the rrnL6 module (Fig 6D). Other clusters of PATs mapped to the 3’-ends of rrnL7, rrnS2, rrnL3b, and rrnS3 modules (Fig 6D). “Internal” clusters throughout the ribosomal RNA genes were also apparent (Fig 6D).

**Fig 7:**
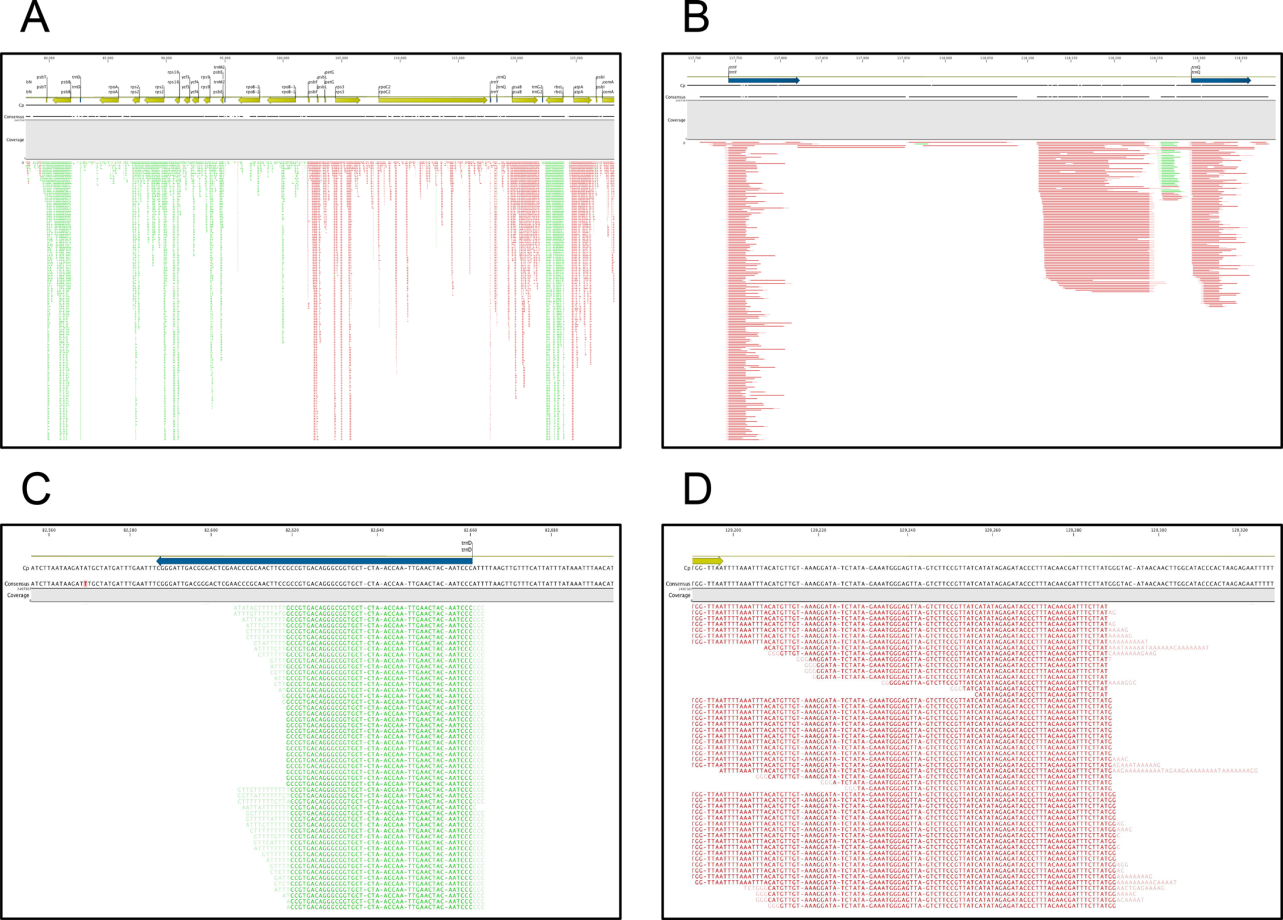
Details of poly(A) tracts for previously described Chlamydomonas chloroplast genes. (A) Genome browser representation of tag mapping to a selected portion of the chloroplast genome that omits the rRNA-encoding regions. The uppermost plots depict details of the gene annotations, the middle line (“consensus”) shows the overall extent of contiguous tag mappings, and the lower plot shows the mappings of individual tags (green or red tics). Red and green tics denote tags oriented in the left-to-right (5’-3’) or right-to-left (5’-3’) directions. (B) Close-up browser view of reads that map to the chloroplast trnD gene. The sequences of the individual reads are visible; lettering in light shaded fonts denote extraneous sequence that does not map to the genome, but rather represents the mixed heteropolymeric tract as noted in the text. (D) Close-up browser view of reads that map to the chloroplast atpH gene. The sequences of the individual reads are visible; lettering in light shaded fonts denote extraneous sequence that does not map to the genome, but rather represents the mixed homopolymer as noted in the text.

Polyadenylation has been noted to affect all classes of RNA in the Chlamydomonas chloroplast [36]; this is corroborated by the data presented in this study. Specifically, while the large majority of PATs that map to the chloroplast genome map to rRNA-encoding regions, there is an abundance of tags that map throughout the chloroplast genome (S9 File). The mapped positions include both the extremities and internal sites of protein-coding genes (e.g., Fig 7A), as well as tRNA loci (an example of two such loci is shown in Fig 7B). Interestingly, most tRNA-associated PATs define 3’-ends that are internal to the mature, full-length tRNA. Importantly, the polyadenylated 3’-ends of atpB, petD, and trnR1 RNAs described in Komine et al. [36] are recapitulated in this study (S6 Fig). The rrn5 3’-ends described in Komine et al. are 10 nts removed from one of the sites seen in this study (S6 Fig). Komine et al. noted a difficulty in generating specific 3’-RACE products for this gene, which may be related to this slight discrepancy. No reads mapped to the trnE1 gene in this study. Taken together, the agreement of the results presented here with those described earlier indicates that the organellar poly(A) sites identified in the current study are accurate and reflect the scope of polyadenylation of Chlamydomonas chloroplast RNAs.

Others have noted that the “poly(A)” tails present on organellar RNAs are often heteropolymeric, with an occasional nucleotide other than A present [37]. Because of the nature of the reverse transcription primer (being anchored in a way that would allow priming at positions within the poly(A) tail that have a non-A base), it is possible to examine the occurrence of such instances. As shown in Fig 7C and 7D, heteropolymeric tracts can indeed be seen; the two examples shown illustrate the phenomenon for a polyadenylated tRNA (Fig 7C) and protein-coding mRNA (Fig 7D).

PATs map to 97 annotated features of the chloroplast genome (S9 File). For more than half (61) of the annotation units, the relative abundances of PATs that map to individual genes changes in significant ways in at least one of the four growth conditions (S10 File). The affected genes include ones that encode ribosomal RNAs, tRNAs, and various proteins. These observations suggest that RNA polyadenylation in the chloroplast is dynamic and may respond to different conditions in a gene- and RNA- specific fashion.

## Discussion

### An updated view of nuclear polyadenylation in Chlamydomonas

The results of the genome-wide determination of poly(A) sites in Chlamydomonas presented here reinforce previous conclusions regarding the process, but also give cause for some revision of prior conclusions. Early studies involving analysis of individual clones and ESTs (e.g., [31]) suggested that the motif UGUAA was a probable polyadenylation signal in Chlamydomonas and other Chlorophyta species, and was analogous to the A-rich “near-upstream element”, or NUE, seen in the polyadenylation signals of higher plants [38]. A more recent study, involving almost 17,000 EST sequences containing poly(A) tracts, confirmed this suggestion, as this motif was found associated with about 52% of confirmed poly(A) sites in Chlamydomonas [30]; this value is similar to that (58%) reported here. Another report involving analysis of large EST, Illumina, and 454 datasets drew similar conclusions, but noted a somewhat lower association of this motif with poly(A) sites [32].

The reports to date (e.g., [30–32, 39]) leave little doubt as to the importance of the UGUAA motif in specifying poly(A) sites in Chlamydomonas. Indeed, if one base substitutions in the motif are allowed, then more than 95% of all sites identified here can be associated with the UGUAA motif. This strongly suggests that poly(A) sites in Chlamydomonas are defined primarily by this motif which stands it apart from higher plants, animals, and yeast. The reasons for these differences are not clear and raise tantalizing possibilities. In animals, recent studies indicate that two core polyadenylation complex subunits, CPSF30 and Wdr33, associate with the AAUAAA motif [40, 41]. While the RNA-binding properties of recombinant Wdr33 have not been studied, CPSF30 by itself has little sequence preference beyond one for U-rich sequences [42]. Thus, the association of these proteins with AAUAAA in the complex probably reflects altered associations or sequence preferences that are due to the combined properties of the complex (that includes RNA binding proteins in addition to these two). Chlamydomonas possesses genes that encode possible orthologs of each of these proteins [43]. However, while the similarity between the Chlamydomonas and plant Wdr33 proteins is high, the putative Chlamydomonas CPSF30 ortholog has very limited sequence similarity with either the plant or animal CPSF30 orthologs. Thus, it is possible that in Chlamydomonas, the CPSF30 protein has evolved preferential affinity for a different and relatively exacting sequence in the polyadenylation signal.

A different parallel suggests an alternative scenario. The so-called CFIm sub-complex in animals includes a 25 kDa subunit, CFIm25. This subunit has a strong preference *in vitro* for RNAs that possess the motif UGUA [44]. This motif is included in the highly-conserved Chlamydomonas polyadenylation signal (UGUAA). Chlamydomonas possesses a probable CFIm25 ortholog that is highly conserved [43]. The similarity in the Chlamydomonas poly(A) signal and preferred CFIm25 binding motif raises the possibility that, in this organism, the CFIm25-RNA contact has replaced other contacts as the primary or sole determinant of poly(A) site choice. Indeed, given the very limited similarity between the suggested Chlamydomonas CPSF30 subunit and its orthologs in other eukaryotes, it is conceivable that the putative Chlamydomonas CPSF30 is in fact not an authentic polyadenylation factor subunit. In this scenario, the function of CPSF30 in the complex would be replaced by additional roles for CFIm25. Of course, all of this is rather speculative, and these alternatives seem far-fetched. Nonetheless, the curious substitution of the A-rich polyadenylation signal with UGUAA raises interesting questions regarding the composition and functioning of the Chlamydomonas polyadenylation complex, and more generally of the evolution of this complex.

### The scope and impact of alternative polyadenylation

The results presented in this report indicate that some 75% of expressed Chlamydomonas genes possess more than one poly(A) site (Fig 2A). However, when nearby sites are grouped into poly(A) site clusters, only some 40% of expressed genes are found to possess more than one such cluster (Fig 2B). This number is somewhat lower than another recent estimate (about 68%; [32]). The reasons for this discrepancy are not clear, although Zhao et al. [32] compiled their data by extracting poly(A)-containing sequences or reads from extant EST, 454, and Illumina RNA-Seq datasets, and not from dedicated poly(A) tags (as done in this report). Zhao et al. suggest that the overall depth of sequence coverage may affect the overall tabulation of poly(A) sites in Chlamydomonas. If this is the source of the discrepancy, then it seems likely that those sites apparently missed in this report are sites that are chosen at very low levels, or that are associated with genes whose expression is low. It is notable that the nucleotide composition profiles for poly(A) sites in this report (Fig 3A) is very similar to sites defined by curated EST and 454 data, but different from that seen in sites derived from poly(A) reads extracted from Illumina RNA-Seq reads (Fig 2 in [32]). This may be an indication that many of the sites identified from extracted Illumina reads are either non-canonical or artifactual. Given this and the consistency seen among the EST- and 454- derived PAC lists in Zhao et al. with the PACs described in this report, it is likely that the true extent of possible alternative polyadenylation (represented by the numbers of genes with more than one site) is lower than the 68% number assigned before, and closer to the value (some 40%) as indicated by the results in this report.

The numbers of genes possessing more than one poly(A) site provides an upper limit (of sorts) of the extent of possible alternative poly(A) site choice, and very likely does not reflect the true extent of such choices. Interestingly, the results presented here (S5 Fig and S8 File) indicate that a very small number of all PACs (at most, some 1.4%) are utilized to different extents under one or more of the four growth conditions used in this study. Moreover, the overwhelming majority of such events involve sites situated in 3’-UTRs, and thus likely do not affect the protein-coding capacity of associated genes. While limited to a relatively small number of different growth conditions, these data nonetheless suggest that true alternative polyadenylation is not extensive in Chlamydomonas, but is likely limited to a small number of sites and genes. This places Chlamydomonas in significant contrast to animals, in which more extensive networks of differentially-utilized poly(A) sites have been documented (e.g., [45, 46]).

As unicellular algae continue to be pursued as production platforms for various biotechnological industries, understanding the critical cellular process of mRNA polyadenylation (in all three compartments) and the implications of it could eliminate undesirable, and potentially costly, oversights during design and implementation phases. For instance, Lumbreras et al. has shown that incorporation of the RbcS2 intron 1 near the promoter of transgene constructs leads to increased levels of transgene expression [47]. However, given that some Chlamydomonas introns carry within them poly(A) sites, blindly selecting intron sequences for inclusion in genetic constructs could have deleterious effects for the desired outcome, as alternative polyadenylation sites within introns could lead to a non-functional protein. With the data set provided here, one could potentially eliminate these unwanted possibilities but still retain the desired benefits of including an intron(s) in the transgene sequence of interest, given that the growth conditions employed matched those of this study.

### Organellar polyadenylation

A number of previous studies have documented the relevance of polyadenylation to organellar RNA homeostasis in Chlamydomonas, linking the process to a destabilization of RNAs [35, 48]. The results presented here provide a global overview of the impact of polyadenylation on RNA metabolism in the chloroplast and mitochondria and offer additional insight into how it might be affecting the status of these organelles.

The general distribution of mapped PATs along the chloroplast genome (Fig 6A) is consistent with earlier results indicating that the three generic classes of RNA–mRNA, tRNA, and rRNA–are all polyadenylated in the Chlamydomonas chloroplast [36]. However, the large majority of chloroplast-associated PATs map to the rRNA-encoding cluster (Fig 6A), and are decidedly non-random in their distribution. In particular, the major rRNA-situated poly(A) sites observed in Fig 6C are internal sites that fall within the 7S and 23S rRNA coding regions. While not exactly coincidental, these sites are reminiscent of the sites of polyadenylation of *Escherichia coli* rRNA breakdown products [49], suggesting that a primary function of polyadenylation in the chloroplast is associated with the turnover of ribosomal RNAs. Interestingly, the fraction of PATs that map to the chloroplast (and primarily to the plastid rRNAs) changes significantly under the different growth conditions with most PATs observed for cells grown in acetate-supplemented media (S2 File). This suggests another parallel with prokaryotes [50], namely that growth status can have a significant impact on ribosome composition and turnover in the *Chlamydomonas* chloroplast.

While the bulk of polyadenylated RNAs in the chloroplast are ribosomal RNAs, polyadenylated RNAs that map to almost every annotated chloroplast gene are also seen (S9 File). The profiles seen in this study extend the known scope of RNA polyadenylation in the Chlamydomonas chloroplast. As chloroplast RNA polyadenylation is typically associated with turnover, these profiles may reflect a complexity in turnover pathways or mechanisms heretofore unrealized. The substantial changes in poly(A) site profiles that are seen in the different growth conditions in this study (S10 File) suggest that, as seems to be the case for ribosomal RNAs, the polyadenylation (and thus degradation) of other classes of chloroplast RNAs is dynamic and responsive to changes in growth and environment.

As is seen in the chloroplast, almost all mitochondrial RNA polyadenylation is associated with ribosomal RNAs (Fig 6B and 6D). However, in the mitochondrion, most of the polyadenylated RNA 3’-ends correspond to the 3’-ends of mature ribosomal RNAs (Fig 6D). The meaning of this is not clear, given that RNA polyadenylation in mitochondria is associated with RNA turnover [35]. The polyadenylation seen at mature 3’-ends may reflect early steps in rRNA turnover; perhaps the mitochondrial rRNA must first be polyadenylated before being broken down. Alternatively, it is possible that ribosomal RNA polyadenylation in the Chlamydomonas mitochondrion is associated with processes apart from turnover. These issues await further experimental study.

## Summary

The results presented in this study confirm the central role of the motif UGUAA as a polyadenylation signal in nuclear genes in *Chlamydomonas reinhardtii*, and indicate that as many as 95% of all poly(A) sites in the organism are controlled by motifs related to UGUAA. In so doing, the conclusions of several earlier reports are confirmed and extended. However, in contrast to earlier studies, the data presented herein suggest at most a limited role for alternative poly(A) site choice in gene expression in Chlamydomonas. This in turn raises the possibility that posttranscriptional control *via* alternative polyadenylation would seem to have limited potential as a tool for manipulating foreign gene expression in the nuclear genome of this organism. Finally, this report provides a detailed genome-wide view of RNA polyadenylation in the two organelles, suggesting both a dynamic and changing contribution of RNA polyadenylation towards gene expression in these compartments and a significant role for polyadenylation in ribosomal RNA metabolism and turnover.

## Supporting Information

S1 Fig Growth stage analysis of Chlamydomonas cultures grown in the four different media types used in this study. A five-day-old culture of Chlamydomonas grown in TP or HS was used to inoculate 100 mL of TP and TAP media or HS and HAS media, respectively. OD values were measured at 750 nm using a NanoDrop spectrophotometer and 2 μL of sample at one-day intervals following inoculation.
(TIF)

S2 Fig Principal component analysis of gene expression for the twelve Chlamydomonas PAT libraries created. Gene expression was determined by mapping trimmed PATs to genes using Bedtools and porting the outcomes into CLC Genomics Workbench. The latter was then used to assess gene expression using the “Empirical Analysis of DGE” tool. Parameters used for this were: Total count filter cutoff = 5.0. Estimate tagwise dispersions = Yes. Comparisons = All pairs. Bonferroni corrected = Yes. FDR corrected = Yes. Common dispersion estimate: 2.4520e-02, coefficient of biological variation: 1.5659e-01. The PCA plot was generated using these results. In the plot, red dots represent TP samples, green dots TAP, blue dots HS, and yellow dots HAS.
(TIF)

S3 Fig Plots of the position-by-position base composition of poly(A) sites that map cannot be linked with recognizable UGUAA motifs. Y-axis values are the fractional nucleotide content at each position (plotted along the x-axis); individual traces are color coded as indicated. On the x-axis, “0” denotes the actual cleavage/polyadenylation site; negative values represent positions 5’ (upstream) of the poly(A) site and positive values are positions 3’ (downstream) of the poly(A) site. The number of sites used to generate this plot was 1,942.
(TIF)

S4 Fig Characteristics of poly(A) sites that map to unannotated genomic positions. (A) Plots of the position-by-position base composition of poly(A) sites that map to unannotated regions of the Chlamydomonas genome. Y-axis values are the fractional nucleotide content at each position (plotted along the x-axis); individual traces are color-coded as indicated. On the x-axis, “0” denotes the actual cleavage/polyadenylation site; negative values represent positions 5’ (upstream) of the poly(A) site and positive values are positions 3’ (downstream) of the poly(A) site. (B) Motif analysis of poly(A) sites that map to unannotated positions in the Chlamydomonas genome. The occurrences of 5 nt motifs was determined using SignalSleuth2 [51]. The relative position of the motif is given on the x-axis, with the poly(A) site being set as “0”. The numerical count of each motif is given on the y-axis. The plot shows the distributions of the 50 most-abundant motifs; these are listed in the legend embedded on the right. For the plots in panels A and B, n = 5,307.
(TIF)

S5 Fig Genome browser representations of genes whose poly(A) site usage varies in one or more of the experimental conditions used for this study. For each plot, a representation of the respective gene is shown at the top. Beneath are illustrations showing the numbers of locations of PATs that map to the respective genes in each of the four treatments. Treatment designations are given on the left. For these plots, the three replicates for each treatment were pooled before mapping. Red tics denote tags oriented in a left-to-right (5’-3’) direction. Green tics represent tags oriented in a right-to-left (5’-3’) orientation. Other colored interruptions in the individual tics represent Illumina sequencing errors.
(TIF)

S6 Fig Close-up genome browser illustrations of mappings to three chloroplast genes whose poly(A) sites have been reported elsewhere (see the text). The general features of the gene representations and tag color coding are as in S5 Fig. Above each gene representation are the positions of poly(A) sites reported in Komine et al. [36], noted with brackets or diamond symbols. Note that, for this figure, the homopolymeric poly(A)/poly(T) tracts present in the PATs have been trimmed (and hence will not be displayed). Also note that, as in Fig 7, heteropolymeric tracts that do not map to the genome are represented as lightly-shaded lettering.
(TIF)

S1 File Computational pipeline used to analyze the sequencing data. (DOCX)

S2 File Summaries of read mapping outcomes for the twelve libraries. (XLSX)

S3 File Results of the gene expression analysis conducted using CLC Genomics Workbench. (XLSX)

S4 File Poly(A) site (PAS) list. (XLSX)

S5 File Poly(A) site Cluster (PAC) list. (XLSX)

S6 File Motif discovery for poly(A) sites that map to 5’-UTRs, introns, and protein-coding regions. (XLSX)

S7 File Annotation features closest to PACs that map to unannotated regions. (XLSX)

S8 File DEXSeq analysis output. (XLSX)

S9 File Lists of PAS for the Chlamydomonas organelles. (XLSX)

S10 File Results of the chloroplast gene expression analysis conducted using CLC Genomics Workbench. (XLSX)

## References

[1] Darnell JE Jr. Reflections on the history of pre-mRNA processing and highlights of current knowledge: a unified picture. RNA. 2013;19(4):443–60. 10.1261/rna.038596.113 23440351 PMC3677254

[2] de Almeida SF, Carmo-Fonseca M. Cotranscriptional RNA checkpoints. Epigenomics. 2010;2(3):449–55. 10.2217/epi.10.21 . 22121903

[3] Lenasi T, Barboric M. Mutual relationships between transcription and pre-mRNA processing in the synthesis of mRNA. Wiley Interdiscip Rev RNA. 2013;4(2):139–54. 10.1002/wrna.1148 . 23184646

[4] Hunt AG. Messenger RNA 3' end formation in plants. Current topics in microbiology and immunology. 2008;326:151–77. Epub 2008/07/18. . 18630752 10.1007/978-3-540-76776-3_9

[5] Tian B, Graber JH. Signals for pre-mRNA cleavage and polyadenylation. Wiley Interdisciplinary Reviews: RNA. 2012;3(3):385–96. Epub 2011/10/21. 10.1002/wrna.116 . 22012871 PMC4451228

[6] Mayr C, Bartel DP. Widespread shortening of 3'UTRs by alternative cleavage and polyadenylation activates oncogenes in cancer cells. Cell. 2009;138(4):673–84. 10.1016/j.cell.2009.06.016 19703394 PMC2819821

[7] Simpson GG, Dijkwel PP, Quesada V, Henderson I, Dean C. FY is an RNA 3' end-processing factor that interacts with FCA to control the Arabidopsis floral transition. Cell. 2003;113(6):777–87. . 12809608 10.1016/s0092-8674(03)00425-2

[8] Harris EH. Chlamydomonas as a Model Organism. Annu Rev Plant Physiol Plant Mol Biol. 2001;52:363–406. 10.1146/annurev.arplant.52.1.363 . 11337403

[9] Silflow CD, Lefebvre PA. Assembly and motility of eukaryotic cilia and flagella. Lessons from Chlamydomonas reinhardtii. Plant Physiol. 2001;127(4):1500–7. 11743094 PMC1540183

[10] Scaife MA, Nguyen GT, Rico J, Lambert D, Helliwell KE, Smith AG. Establishing Chlamydomonas reinhardtii as an industrial biotechnology host. Plant J. 2015;82(3):532–46. 10.1111/tpj.12781 25641561 PMC4515103

[11] Scranton MA, Ostrand JT, Fields FJ, Mayfield SP. Chlamydomonas as a model for biofuels and bio-products production. Plant J. 2015;82(3):523–31. 10.1111/tpj.12780 . 25641390 PMC5531182

[12] Torzillo G, Scoma A, Faraloni C, Giannelli L. Advances in the biotechnology of hydrogen production with the microalga Chlamydomonas reinhardtii. Crit Rev Biotechnol. 2014 10.3109/07388551.2014.900734 . 24754449

[13] Rasala BA, Muto M, Lee PA, Jager M, Cardoso RM, Behnke CA, et al Production of therapeutic proteins in algae, analysis of expression of seven human proteins in the chloroplast of Chlamydomonas reinhardtii. Plant Biotechnol J. 2010;8(6):719–33. 10.1111/j.1467-7652.2010.00503.x 20230484 PMC2918638

[14] Rasala BA, Muto M, Sullivan J, Mayfield SP. Improved heterologous protein expression in the chloroplast of Chlamydomonas reinhardtii through promoter and 5' untranslated region optimization. Plant Biotechnol J. 2011;9(6):674–83. 10.1111/j.1467-7652.2011.00620.x . 21535358

[15] Mussgnug JH. Genetic tools and techniques for Chlamydomonas reinhardtii. Appl Microbiol Biotechnol. 2015;99(13):5407–18. 10.1007/s00253-015-6698-7 . 26025017

[16] Jinkerson RE, Jonikas MC. Molecular techniques to interrogate and edit the Chlamydomonas nuclear genome. Plant J. 2015;82(3):393–412. 10.1111/tpj.12801 . 25704665

[17] Rasala BA, Lee PA, Shen Z, Briggs SP, Mendez M, Mayfield SP. Robust expression and secretion of Xylanase1 in Chlamydomonas reinhardtii by fusion to a selection gene and processing with the FMDV 2A peptide. PLoS One. 2012;7(8):e43349 10.1371/journal.pone.0043349 22937037 PMC3427385

[18] Ohme-Takagi M, Taylor CB, Newman TC, Green PJ. The effect of sequences with high AU content on mRNA stability in tobacco. Proceedings of the National Academy of Sciences of the United States of America. 1993;90(24):11811–5. 8265631 10.1073/pnas.90.24.11811 PMC48074

[19] De Rocher EJ, Vargo-Gogola TC, Diehn SH, Green PJ. Direct evidence for rapid degradation of Bacillus thuringiensis toxin mRNA as a cause of poor expression in plants. Plant Physiol. 1998;117(4):1445–61. 9701600 10.1104/pp.117.4.1445 PMC34908

[20] Diehn SH, Chiu WL, De Rocher EJ, Green PJ. Premature polyadenylation at multiple sites within a Bacillus thuringiensis toxin gene-coding region. Plant Physiol. 1998;117(4):1433–43. 9701599 10.1104/pp.117.4.1433 PMC34907

[21] Diehn SH, De Rocher EJ, Green PJ. Problems that can limit the expression of foreign genes in plants: lessons to be learned from B.t. toxin genes. Genet Eng (N Y). 1996;18:83–99. . 8785128 10.1007/978-1-4899-1766-9_6

[22] Iannacone R, Grieco PD, Cellini F. Specific sequence modifications of a cry3B endotoxin gene result in high levels of expression and insect resistance. Plant Mol Biol. 1997;34(3):485–96. . 9225859 10.1023/a:1005876323398

[23] Gorman DS, Levine RP. Cytochrome f and plastocyanin: their sequence in the photosynthetic electron transport chain of Chlamydomonas reinhardi. Proceedings of the National Academy of Sciences of the United States of America. 1965;54(6):1665–9. 4379719 10.1073/pnas.54.6.1665 PMC300531

[24] Sueoka N. Mitotic Replication of Deoxyribonucleic Acid in Chlamydomonas Reinhardi. Proceedings of the National Academy of Sciences of the United States of America. 1960;46(1):83–91. 16590601 10.1073/pnas.46.1.83 PMC285018

[25] Ma L, Pati PK, Liu M, Li QQ, Hunt AG. High throughput characterizations of poly(A) site choice in plants. Methods. 2014;67(1):74–83. 10.1016/j.ymeth.2013.06.037 23851255 PMC3900603

[26] Pati PK, Ma L, Hunt AG. Genome-wide determination of poly(A) site choice in plants. Methods Mol Biol. 2015;1255:159–74. 10.1007/978-1-4939-2175-1_14 . 25487212

[27] Wang Y, Stessman DJ, Spalding MH. The CO2 concentrating mechanism and photosynthetic carbon assimilation in limiting CO2: how Chlamydomonas works against the gradient. Plant J. 2015;82(3):429–48. 10.1111/tpj.12829 . 25765072

[28] Sherstnev A, Duc C, Cole C, Zacharaki V, Hornyik C, Ozsolak F, et al Direct sequencing of Arabidopsis thaliana RNA reveals patterns of cleavage and polyadenylation. Nature structural & molecular biology. 2012;19(8):845–52. Epub 2012/07/24. 10.1038/nsmb.2345 22820990 PMC3533403

[29] Wu X, Liu M, Downie B, Liang C, Ji G, Li QQ, et al Genome-wide landscape of polyadenylation in Arabidopsis provides evidence for extensive alternative polyadenylation. Proceedings of the National Academy of Sciences, USA. 2011;108(30):12533–8. 10.1073/pnas.1019732108 . PMC3145732 21746925

[30] Shen Y, Liu Y, Liu L, Liang C, Li QQ. Unique features of nuclear mRNA poly(A) signals and alternative polyadenylation in Chlamydomonas reinhardtii. Genetics. 2008;179(1):167–76. 10.1534/genetics.108.088971 18493049 PMC2390596

[31] Wodniok S, Simon A, Glockner G, Becker B. Gain and loss of polyadenylation signals during evolution of green algae. BMC Evol Biol. 2007;7:65 10.1186/1471-2148-7-65 17442103 PMC1868727

[32] Zhao Z, Wu X, Kumar PK, Dong M, Ji G, Li QQ, et al Bioinformatics analysis of alternative polyadenylation in green alga Chlamydomonas reinhardtii using transcriptome sequences from three different sequencing platforms. G3 (Bethesda). 2014;4(5):871–83. 10.1534/g3.114.010249 24626288 PMC4025486

[33] Wu X, Gaffney B, Hunt AG, Li QQ. Genome-wide determination of poly(A) sites in Medicago truncatula: evolutionary conservation of alternative poly(A) site choice. BMC Genomics. 2014;15:615 10.1186/1471-2164-15-615 25048171 PMC4117952

[34] Anders S, Reyes A, Huber W. Detecting differential usage of exons from RNA-seq data. Genome Res. 2012;22(10):2008–17. 10.1101/gr.133744.111 22722343 PMC3460195

[35] Schuster G, Stern D. RNA polyadenylation and decay in mitochondria and chloroplasts. Prog Mol Biol Transl Sci. 2009;85:393–422. 10.1016/S0079-6603(08)00810-6 . 19215778

[36] Komine Y, Kwong L, Anguera MC, Schuster G, Stern DB. Polyadenylation of three classes of chloroplast RNA in Chlamydomonas reinhadtii. RNA. 2000;6(4):598–607. 10786850 10.1017/s1355838200992252 PMC1369940

[37] Zimmer SL, Schein A, Zipor G, Stern DB, Schuster G. Polyadenylation in Arabidopsis and Chlamydomonas organelles: the input of nucleotidyltransferases, poly(A) polymerases and polynucleotide phosphorylase. Plant J. 2009;59(1):88–99. 10.1111/j.1365-313X.2009.03853.x . 19309454

[38] Loke JC, Stahlberg EA, Strenski DG, Haas BJ, Wood PC, Li QQ. Compilation of mRNA polyadenylation signals in Arabidopsis revealed a new signal element and potential secondary structures. Plant Physiol. 2005;138(3):1457–68. . 15965016 10.1104/pp.105.060541 PMC1176417

[39] Li XQ, Du D. Motif types, motif locations and base composition patterns around the RNA polyadenylation site in microorganisms, plants and animals. BMC Evol Biol. 2014;14:162 10.1186/s12862-014-0162-7 25052519 PMC4360255

[40] Chan SL, Huppertz I, Yao C, Weng L, Moresco JJ, Yates JR 3rd, et al CPSF30 and Wdr33 directly bind to AAUAAA in mammalian mRNA 3' processing. Genes & development. 2014;28(21):2370–80. 10.1101/gad.250993.114 25301780 PMC4215182

[41] Schonemann L, Kuhn U, Martin G, Schafer P, Gruber AR, Keller W, et al Reconstitution of CPSF active in polyadenylation: recognition of the polyadenylation signal by WDR33. Genes & development. 2014;28(21):2381–93. 10.1101/gad.250985.114 25301781 PMC4215183

[42] Barabino SM, Hubner W, Jenny A, Minvielle-Sebastia L, Keller W. The 30-kD subunit of mammalian cleavage and polyadenylation specificity factor and its yeast homolog are RNA-binding zinc finger proteins. Genes & development. 1997;11(13):1703–16. . 9224719 10.1101/gad.11.13.1703

[43] Hunt AG, Xing D, Li QQ. Plant polyadenylation factors: conservation and variety in the polyadenylation complex in plants. BMC Genomics. 2012;13:641 10.1186/1471-2164-13-641 23167306 PMC3538716

[44] Brown KM, Gilmartin GM. A mechanism for the regulation of pre-mRNA 3' processing by human cleavage factor Im. Mol Cell. 2003;12(6):1467–76. . 14690600 10.1016/s1097-2765(03)00453-2

[45] Tian B, Manley JL. Alternative cleavage and polyadenylation: the long and short of it. Trends Biochem Sci. 2013;38(6):312–20. 10.1016/j.tibs.2013.03.005 23632313 PMC3800139

[46] Ulitsky I, Shkumatava A, Jan CH, Subtelny AO, Koppstein D, Bell GW, et al Extensive alternative polyadenylation during zebrafish development. Genome Research. 2012;22(10):2054–66. 10.1101/gr.139733.112 22722342 PMC3460199

[47] Lumbreras V, Stevens DR, Purton S. Efficient foreign gene expression in Chlamydomonas reinhardtii mediated by an endogenous intron. The Plant Journal. 1998;14(4):441–7. 10.1046/j.1365-313X.1998.00145.x

[48] Lange H, Sement FM, Canaday J, Gagliardi D. Polyadenylation-assisted RNA degradation processes in plants. Trends Plant Sci. 2009;14(9):497–504. 10.1016/j.tplants.2009.06.007 . 19716749

[49] Maes A, Gracia C, Hajnsdorf E, Regnier P. Search for poly(A) polymerase targets in E. coli reveals its implication in surveillance of Glu tRNA processing and degradation of stable RNAs. Mol Microbiol. 2012;83(2):436–51. 10.1111/j.1365-2958.2011.07943.x . 22142150

[50] Maivali U, Paier A, Tenson T. When stable RNA becomes unstable: the degradation of ribosomes in bacteria and beyond. Biol Chem. 2013;394(7):845–55. 10.1515/hsz-2013-0133 . 23612597

[51] Wu X, Ji G, Li QQ. Computational analysis of plant polyadenylation signals. Methods Mol Biol. 2015;1255:3–11. 10.1007/978-1-4939-2175-1_1 . 25487199

