# Supplementary figures and images for: Experimental Genome-Wide Determination of RNA Polyadenylation in Chlamydomonas reinhardtii
Source: PLoS One. 2016 Jan 5;11(1):e0146107. doi: 10.1371/journal.pone.0146107 (PMC4701671; doi:10.1371/journal.pone.0146107)

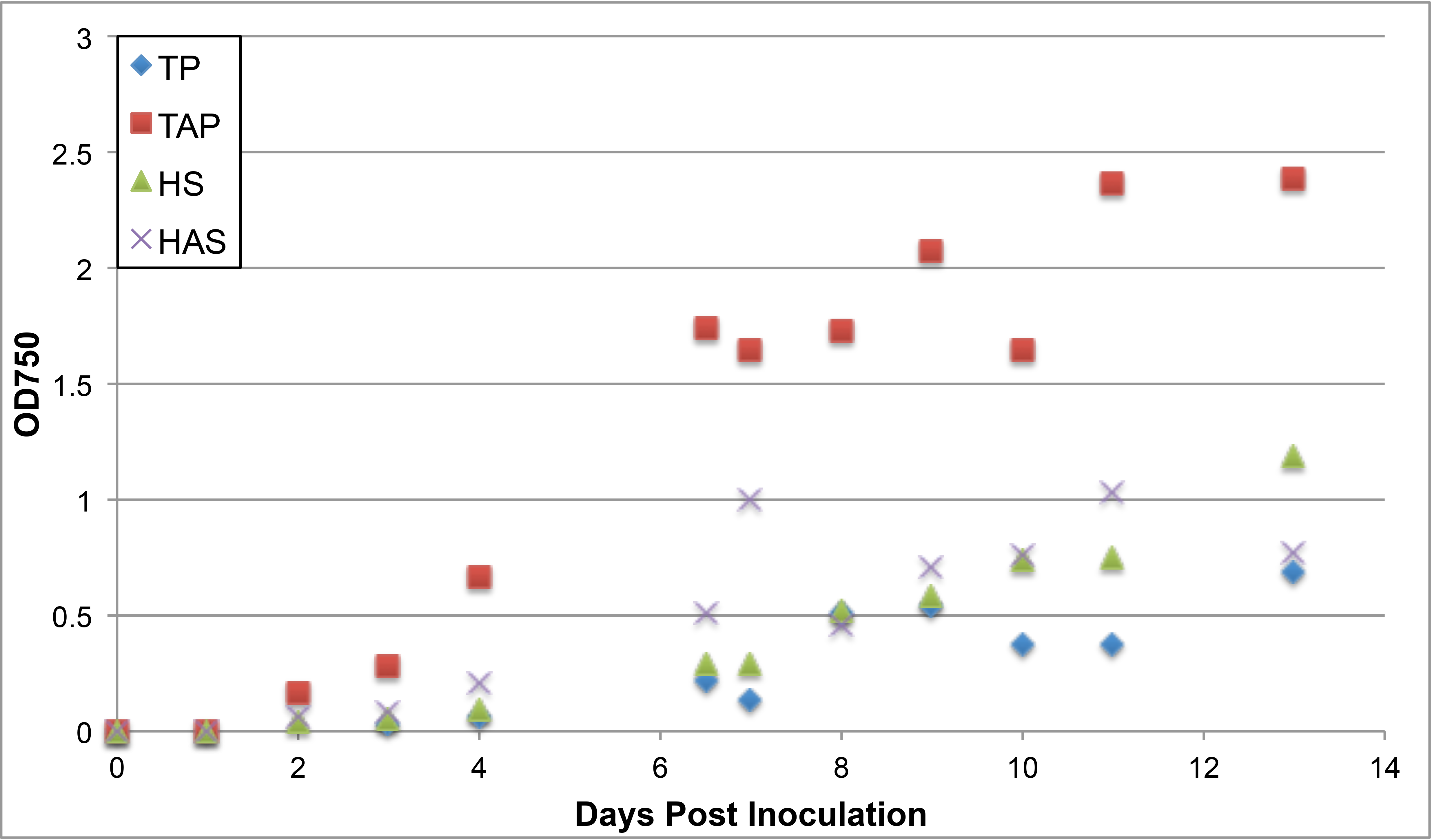

Supplement: S1 Fig — A five-day-old culture of Chlamydomonas grown in TP or HS was used to inoculate 100 mL of TP and TAP media or HS and HAS media, respectively. OD values were measured at 750 nm using a NanoDrop spectrophotometer and 2 μL of sample at one-day intervals following inoculation. (TIF) [file pone.0146107.s001.tif]

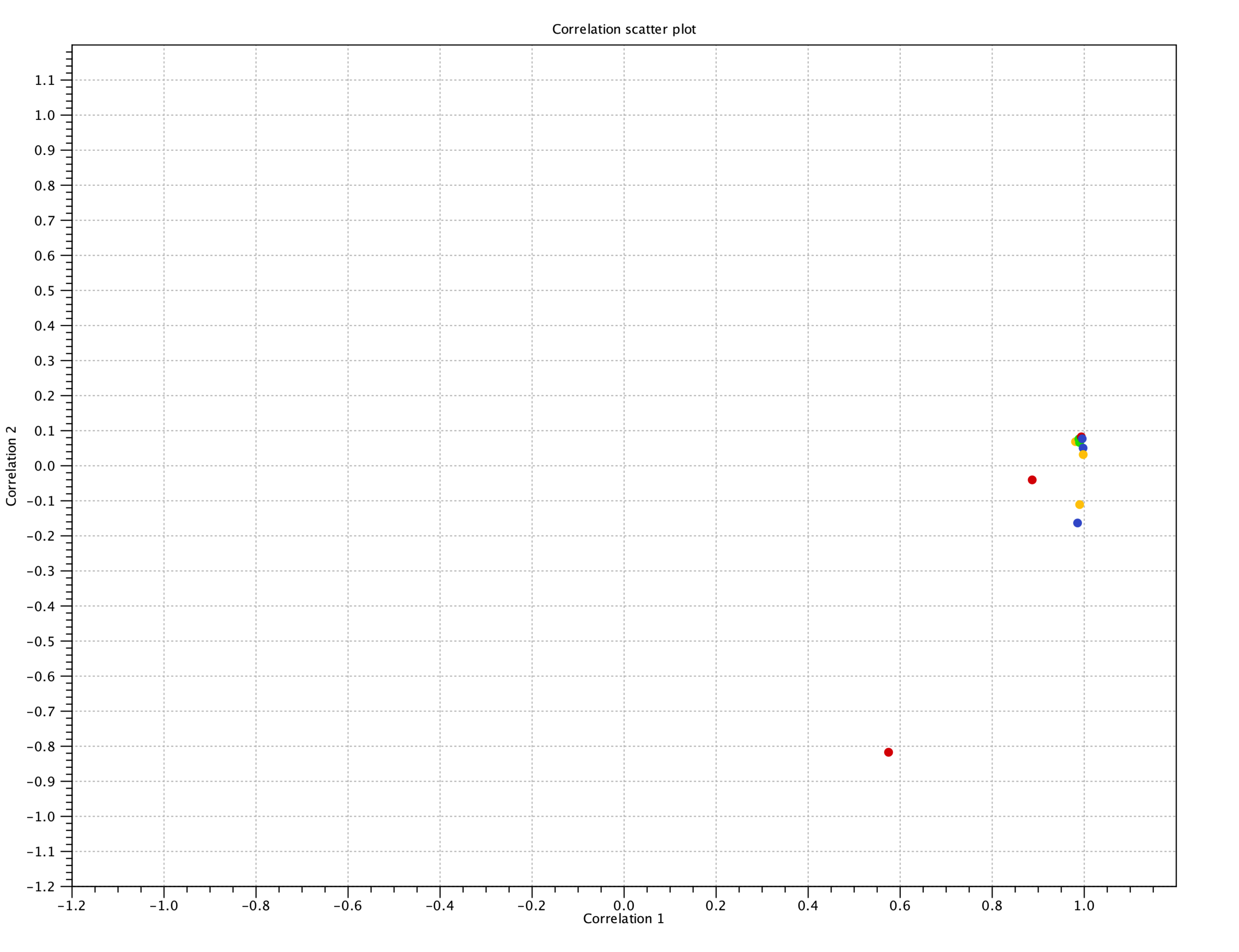

Supplement: S2 Fig — Gene expression was determined by mapping trimmed PATs to genes using Bedtools and porting the outcomes into CLC Genomics Workbench. The latter was then used to assess gene expression using the “Empirical Analysis of DGE” tool. Parameters used for this were: Total count filter cutoff = 5.0. Estimate tagwise dispersions = Yes. Comparisons = All pairs. Bonferroni corrected = Yes. FDR corrected = Yes. Common dispersion estimate: 2.4520e-02, coefficient of biological variation: 1.5659e-01. The PCA plot was generated using these results. In the plot, red dots represent TP samples, green dots TAP, blue dots HS, and yellow dots HAS. (TIF) [file pone.0146107.s002.tif]

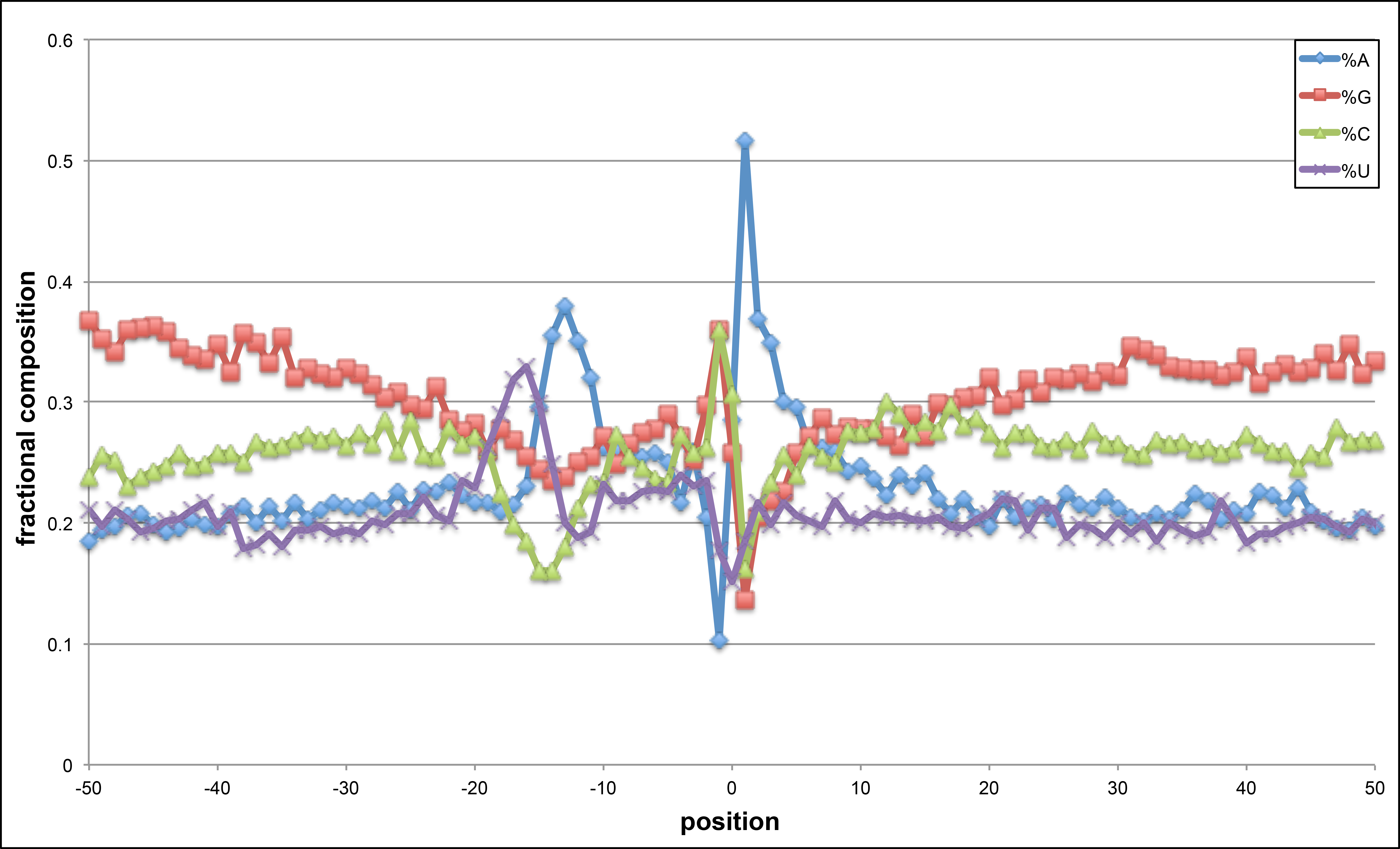

Supplement: S3 Fig — Y-axis values are the fractional nucleotide content at each position (plotted along the x-axis); individual traces are color coded as indicated. On the x-axis, “0” denotes the actual cleavage/polyadenylation site; negative values represent positions 5’ (upstream) of the poly(A) site and positive values are positions 3’ (downstream) of the poly(A) site. The number of sites used to generate this plot was 1,942. (TIF) [file pone.0146107.s003.tif]

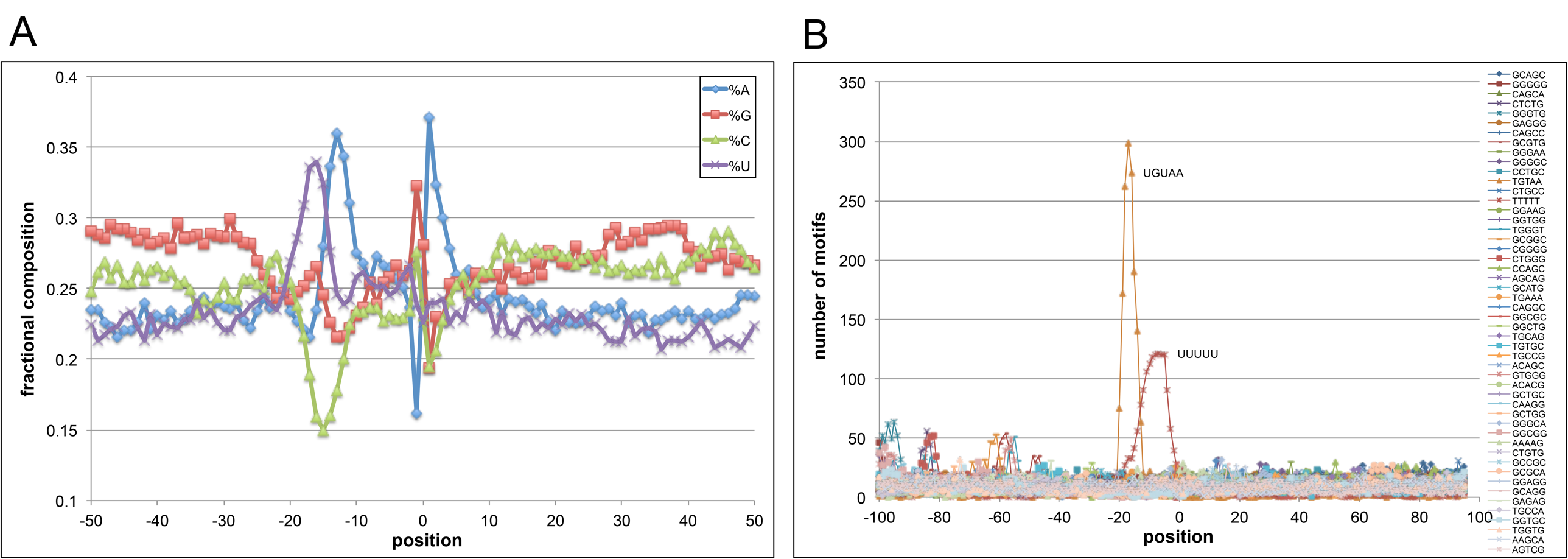

Supplement: S4 Fig — (A) Plots of the position-by-position base composition of poly(A) sites that map to unannotated regions of the Chlamydomonas genome. Y-axis values are the fractional nucleotide content at each position (plotted along the x-axis); individual traces are color-coded as indicated. On the x-axis, “0” denotes the actual cleavage/polyadenylation site; negative values represent positions 5’ (upstream) of the poly(A) site and positive values are positions 3’ (downstream) of the poly(A) site. (B) Motif analysis of poly(A) sites that map to unannotated positions in the Chlamydomonas genome. The occurrences of 5 nt motifs was determined using SignalSleuth2 [51]. The relative position of the motif is given on the x-axis, with the poly(A) site being set as “0”. The numerical count of each motif is given on the y-axis. The plot shows the distributions of the 50 most-abundant motifs; these are listed in the legend embedded on the right. For the plots in panels A and B, n = 5,307. (TIF) [file pone.0146107.s004.tif]

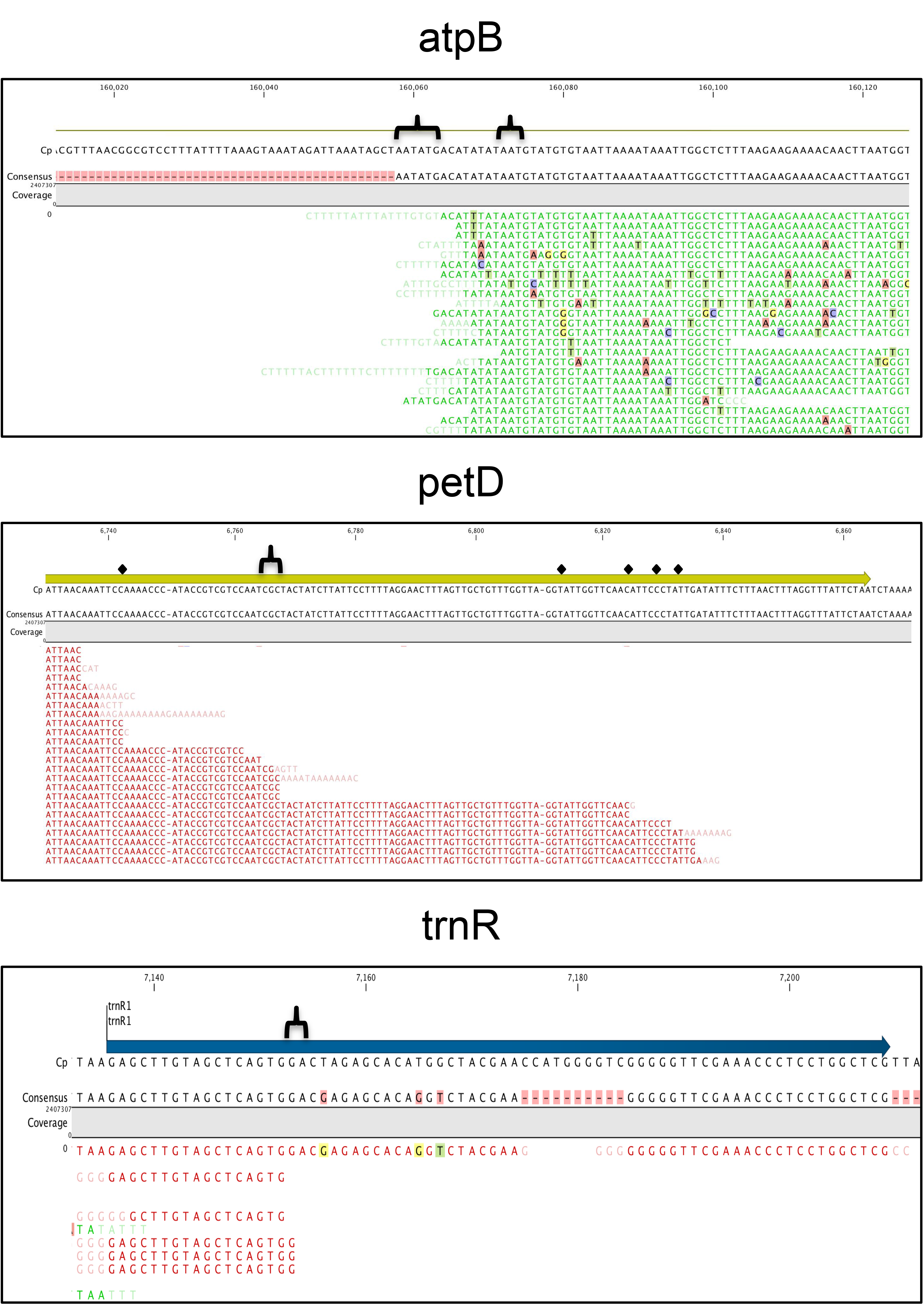

Supplement: S6 Fig — The general features of the gene representations and tag color coding are as in S5 Fig. Above each gene representation are the positions of poly(A) sites reported in Komine et al. [36], noted with brackets or diamond symbols. Note that, for this figure, the homopolymeric poly(A)/poly(T) tracts present in the PATs have been trimmed (and hence will not be displayed). Also note that, as in Fig 7, heteropolymeric tracts that do not map to the genome are represented as lightly-shaded lettering. (TIF) [file pone.0146107.s006.tif]
