# Supplementary material for: Experimental Genome-Wide Determination of RNA Polyadenylation in Chlamydomonas reinhardtii
Source: PLoS One. 2016 Jan 5;11(1):e0146107. doi: 10.1371/journal.pone.0146107 (PMC4701671; doi:10.1371/journal.pone.0146107)
Supplement: S1 File — (DOCX) [file pone.0146107.s007.docx]

**Supplemental File 1 - Poly(A) Tag (PAT) analysis pipeline**

The following describes the various computational steps used in this project for accessing and analyzing the poly(A) tags generated for this project. All steps may be executed on a laptop or desktop computer, and involve the use of commercial software (CLC Genomics Workbench – Qiagen – and Microsoft Excel) as well as freely-available command-line tools run in a Unix framework.

To begin with, it is helpful to review the generic structure of the poly(A) tags generated using Method 2A from Ma et al. [1]. These tags were prepared using reverse transcriptase and primers with the following composition:

5’- (Illumina-compatible sequence) – NNXXXT(18)VN

where NN is a random dinucleotide, XXX is a three-base bar code, and NV a two-base anchor to place the primer at the poly(A)-mRNA junction. The primers used for this project are listed in the table at the end of this document. After sequencing, the first bases read will be the “NN”, followed by the bar code, the oligo-dT tract, and finally the cDNA sequence.

A. Demultiplex and trim

The raw data, downloaded in fastq format (available in Bioproject [PRJNA294481](http://www.ncbi.nlm.nih.gov/bioproject/PRJNA294481)), were imported into the .clc file format for use with CLC Genomics Workbench. For this, the Illumina import option was used, keeping all of the default settings. Individual sequencing samples were extracted from these files using the Demultiplex Reads option. For this, a two nt linker, followed by the three nt bar code appended to a tract of four T’s, was used. (e.g., the bar code used in the tool will be “XXXTTTT”.) The result of this was twelve files defined by the individual bar codes; note that this process removes the linker and bar codes. These files were then trimmed using the Trim Sequences tool; the Trim Library consists of two sequences – the Illumina PE2 sequence (to remove any Illumina adapter sequences from the 3’-end of the PATs; see the table at the end of the file for this sequence) and the sequence TTTTTTTTTT (to remove the oligo-dT tract that remains at the 5’-end of the PATs after demultiplexing). What remains were just the cDNA sequences; for each PAT, the 5’-most base corresponds to the 3’ end of the complementary mRNA.

B. Mapping PATs to the Chlamydomonas genome

The demultiplexed and trimmed PATs were then mapped to the Chlamydomonas genome using the Map Reads to reference tool. For this, the Creinhardtii_281_v5.0 version of the genome was used. The associated annotation file for subsequent work was Creinhardtii_281_v5.5.gene_exons.gff3; both genome and annotation files were downloaded from <http://genome.jgi.doe.gov/> [2]. For the mapping, the following parameters were used:

Cost of insertions and deletions = Linear gap cost

Insertion cost = 3

Deletion cost = 3

Insertion open cost = 6

Insertion extend cost = 3

Deletion open cost = 6

Deletion extend cost = 3

Length fraction = 0.9

Similarity fraction = 0.8

Global alignment = No

Non-specific match handling = Map randomly

Output mode = Create stand-alone read mappings

Create report = Yes

Collect un-mapped reads = No

In addition, genomic positions corresponding to tracts of 6 or more A’s were masked; this was done to eliminate possible instances of internal priming by reverse transcriptase.

These mappings were used to generate variations of the genome browser views presented in the figures in this report. In addition, the results for each bar code (as well as for mappings in which all demultiplexed and trimmed sequences were pooled and used) were exported in bam file format for subsequent processing as described in the following.

B. Creating a master list of mapped and trimmed PATs

Unless otherwise indicated, these steps were performed using the Bedtools suite of programs [3]. When completed, this will generate a complete list of mapped tags reduced to the genomic coordinates that correspond to the 3’ ends of the individual mRNAs.

1. Convert to bed file:

.bamtobed -i <input bam file> ><output file, .bed format>

2. Trim tags to poly(A) sites – this step utilizes a custom tool that converts the chromosomal coordinates for the mapped reads to one nt tags:

tagtrim <input file, bed format (from step B.1)> <output file, bed format>

(This tools – tagtrim – can be obtained upon request – contact Dr. Hunt at [aghunt00@uky.edu](mailto:aghunt00@uky.edu) .)

3. Sort trimmed tags

.sortbed -i <input file, trimmed tags from step B.2, bed format> ><output file, bed format>

C. Make a master Poly(A) Site (PAS) list

These steps will use the complete list of mapped tags to create a list of individual poly(A) sites, along with other information. For this, the results of mapping the pooled libraries were used, so as to create a list of sites present in at least one of the four experimental samples.

1. Make a list of individual poly(A) sites (PAS) along with the PAT numbers for each site:

.groupby -i <input file, trimmed sorted tags from step B.3, bed format> -g 1,2,3,6 -c 2 -o count ><output file, txt format>

2. Convert the output from step C.1 to bed file format. While doing this, also filter out PAS that possess fewer than a set number of tags (nominally, somewhere between 10 and 50, depending on the size of the tag dataset).

a. Sort the txt file from step C.1 file using the UNIX sort command:

sort -k 5n <input file, txt format> ><output file, txt format>

b. Using a text editor (such as TextEdit on a Mac), delete rows corresponding to PATs fewer than the desired number; unless you keep everything, even removing PAS with only 1 tag should reduce the file to a size such that it can be opened in Excel. Save as a txt file.

c. The output from C.2.b will have five columns. The first three are the chromosome number and start and end positions that define the poly(A) sites. The fourth column will be the actual number of PATs that define the poly(A) site, and the fifth column will be the strand that corresponds to the PATs. [NOTE that this strand is the opposite strand of that which “contains:” the gene associated with the poly(A) site; this is because the PATs are complementary to the actual mRNA.] Add a column to this output from C2.2.b with a place marker (.) to yield the following bed file format (Excel was used, but any text editor will suffice):

Chr1 72 73 . 1 +

Save as a bed file. This file will consist of a sorted list of poly(A) sites that includes the PAT numbers seen in the entire experiment.

3. Revise/annotate the PAS list – the idea is to attach gene ID’s and genomic regions (CDS, intron, etc.) to each PAS.

mapbed -S -c 3,9 -o collapse -a <input file – the output from C.2.c> -b <Cre281regionsrev.gff> ><output file, txt format>

Here, use a modified gff file that is based on the Creinhardtii_281_v5.5.gene_exons.gff3 file available from Phytozome.org, but in which all 3’ UTRs have been extended by 25 nts, and that has only gene regions (5’UTR, CDS, intron, 3’UTR) and no other annotations. The reason for the extension is to more fully map PATs to genes and regions that are not properly annotated at their 3’ ends.

4. In Excel, rearrange the columns to get to a bed formatted file: I replace the “.” column of the output from C.3 with gene IDs (from column 9 in the gff file) and the tag numbers with genomic regions (from column 3 in the gff file):

Chr1 start end geneID region (intron, 3’UTR, etc.) strand (“+” or “-“)

After sorting (on the first two columns), save as a text-formatted bed file.

D. Create a master list of poly(A) clusters (PACs)

1. Beginning with the output from step B.3, create a PAC list – for this, use the spacing criteria described in Wu et al. [4]:

mergebed -s -d 24 -c 2,6 -o count,distinct -i <input.bed>><output.txt>

2. Convert the output from step D.1 to bed file format:

The output from step 4 has five columns:

Chr1 72 73 1 +

In Excel, add a column with a place marker (.):

Chr1 72 73 . 1 +

Then sort on column 5 (this is the column that has the tag number for each PAC) and delete rows with fewer than a set limit (10 PATs). Save as a text file using the .bed suffix.

3. Revise/annotate the PAC list – the idea is to attach gene ID’s and genomic regions (CDS, intron, etc.) to each PAC:

mapbed -S -c 3,9 -o collapse -a <input file in bed format> -b <Cre281regionsrev.gff >stability_PAC24_PAT20_region2

Here, you use a modified gff file in which all 3’ UTRs have been extended by 120 nts (as per Wu et al.), and that has only gene regions (5’UTR, CDS, intron, 3’UTR) and no other annotations. In Excel, rearrange the columns to get to .bed format – I replace the “*” column with gene IDs (from column 9 in the gff file) and the tag numbers with genomic regions (from column 3 in the gff file):

Chr1 5737 5980 Parent=AT1G01010.1 three_prime_UTR -

Also, sort the file on columns A and B (smaller to larger). To help keep track of things, it is helpful to include the term “annotated” somewhere in the file name to indicate what has been done. Save in Windows text format, change the suffix to .bed.

E. Run analyses.

1. Determine the PAT frequency for each PAC in the file from step 6 or each PAS in the file from step 9.

./annotatebed -s -counts -i stability_PAC24_PAT20_annotated.bed -files C0trimsort.bed C15trimsort.bed C30trimsort.bed C60trimsort.bed C120trimsort.bed >stabilityregions.txt

here, the –files are the individual sample bed files, trimmed and sorted as in part A. The output is a list of each PAC, with columns that tell the numbers of tags in each individual sample that map to the PAC.

Note that, here, you use lower case “s”. This is because, until now, PATs are antisense to genomic (TAIR-derived) annotations. However, when you make the annotated PAC file, you retain the orientation of the PAC, not the genomic annotation. Thus, PACs and PATs have the same orientation.

2. Determine PAT frequency for each gene

for this, use a special gff file (TAIR10genes120.gff ) that has only Arabidopsis genes. In this file, the genes have been extended by 120 nts on their 3’ ends (as was the case with 3’-UTRs above).

./annotatebed -S -counts -i TAIR10genes120.gff -files C0trimsort.bed C15trimsort.bed C30trimsort.bed C60trimsort.bed C120trimsort.bed >stabilitygenes.txt

Note the use of upper case “S” here. This gives a list that may be used for gene-level expression analysis or other purposes.

3. Gene expression analysis using CLC Genomic Workbench

The output from Part E.2 was converted to one usable for CLC Genomics Workbench; briefly, this involved removing all but the Gene Identifier and individual tag number columns (using Excel), adding a header row with titles for each column (Gene_ID TP1 TP2 etc..), and saving in csv format. These data were imported into CLC Genomics Workbench and gene expression determined using the “Empirical Analysis of DGE” tool, with a total filter cut-off of 5. The results are summarized in S3_File.

**ONE FINAL NOTE:** You should not copy and paste the command line commands from this file into Terminal, since Word formats some characters (such as “-“) in ways that Unix and/or Bedtools mis-handles. Create a text-only file (with TextEdit, for example) and prepare your commands in this file.

| Primer name | Sample | sequence |
| --- | --- | --- |
| RT-PE3a |  | ACACTCTTTCCCTACACGACGCTCTTCCGATCTNNCCCTTTTTTTTTTTTTTTTTTVN |
| RT-PE3b |  | ACACTCTTTCCCTACACGACGCTCTTCCGATCTNNCGGTTTTTTTTTTTTTTTTTTVN |
| RT-PE3c |  | ACACTCTTTCCCTACACGACGCTCTTCCGATCTNNAACTTTTTTTTTTTTTTTTTTVN |
| RT-PE3d |  | ACACTCTTTCCCTACACGACGCTCTTCCGATCTNNAGCTTTTTTTTTTTTTTTTTTVN |
| RT-PE3e |  | ACACTCTTTCCCTACACGACGCTCTTCCGATCTNNACGTTTTTTTTTTTTTTTTTTVN |
| RT-PE3f |  | ACACTCTTTCCCTACACGACGCTCTTCCGATCTNNAGATTTTTTTTTTTTTTTTTTVN |
| RT-PE3g |  | ACACTCTTTCCCTACACGACGCTCTTCCGATCTNNCCGTTTTTTTTTTTTTTTTTTVN |
| RT-PE3h |  | ACACTCTTTCCCTACACGACGCTCTTCCGATCTNNCAATTTTTTTTTTTTTTTTTTVN |
| RT-PE3j |  | ACACTCTTTCCCTACACGACGCTCTTCCGATCTNNCAGTTTTTTTTTTTTTTTTTTVN |
| RT-PE3k |  | ACACTCTTTCCCTACACGACGCTCTTCCGATCTNNGGATTTTTTTTTTTTTTTTTTVN |
| RT-PE3l |  | ACACTCTTTCCCTACACGACGCTCTTCCGATCTNNCCATTTTTTTTTTTTTTTTTTVN |
| RT-PE3m |  | ACACTCTTTCCCTACACGACGCTCTTCCGATCTNNCACTTTTTTTTTTTTTTTTTTVN |
| PE2 |  | CAAGCAGAAGACGGCATACGAGATCGGTCTCGGCATTCCTGCTGAACCGCTCTTCCGATCT |

References for this file

1. Ma L, Pati PK, Liu M, Li QQ, Hunt AG. High throughput characterizations of poly(A) site choice in plants. Methods. 2014;67(1):74-83. doi: 10.1016/j.ymeth.2013.06.037. PubMed PMID: 23851255; PubMed Central PMCID: PMC3900603.

2. Nordberg H, Cantor M, Dusheyko S, Hua S, Poliakov A, Shabalov I, et al. The genome portal of the Department of Energy Joint Genome Institute: 2014 updates. Nucleic Acids Res. 2014;42(Database issue):D26-31. doi: 10.1093/nar/gkt1069. PubMed PMID: 24225321; PubMed Central PMCID: PMCPMC3965075.

3. Quinlan AR, Hall IM. BEDTools: a flexible suite of utilities for comparing genomic features. Bioinformatics. 2010;26(6):841-2. doi: 10.1093/bioinformatics/btq033. PubMed PMID: 20110278; PubMed Central PMCID: PMCPMC2832824.

4. Wu X, Liu M, Downie B, Liang C, Ji G, Li QQ, et al. Genome-wide landscape of polyadenylation in Arabidopsis provides evidence for extensive alternative polyadenylation. Proceedings of the National Academy of Sciences, USA. 2011;108(30):12533-8. doi: 10.1073/pnas.1019732108. PubMed PMID: ISI:000293129900066.
